# Anti-Neuroinflammatory Naphtho-*γ*-Pyrones from a Deep-Sea-Derived Fungus *Aspergillus niger* 3A00562

**DOI:** 10.3390/md24040125

**Published:** 2026-03-27

**Authors:** Zi-Han Xu, Zheng-Biao Zou, Chun-Xiu Wang, Chen Li, Xian-Wen Yang, Jun-Song Wang

**Affiliations:** 1 Center for Molecular Metabolism, School of Environmental and Biological Engineering, Nanjing University of Science and Technology, 200 Xiaolingwei Street, Nanjing 210094, China; xuzihan@njust.edu.cn; 2 School of Basic Medicine and Life Science, Hainan Academy of Medical Sciences, Hainan Medical University, 3 Xueyuan Road, Haikou 571199, China; zouzhengbiao@muhn.edu.cn (Z.-B.Z.); w2326875992@163.com (C.-X.W.); hy0211100@muhn.edu.cn (C.L.)

**Keywords:** marine natural products, proteomics, neuroprotection, anti-neuroinflammatory, NOS2

## Abstract

Inhibition of inflammation and oxidative stress is increasingly recognized as a promising therapeutic strategy for neurodegenerative diseases. In this study, we isolated two new dimeric naphtho-*γ*-pyrone (a*S*)-fonsecinones B and D (**1** and **2**) and 14 known compounds (**3**–**16**) from the deep-sea-derived fungus *Aspergillus niger* 3A00562. Their structures were unambiguously determined through integrated physicochemical and spectroscopic analyses. Screening for neuroinflammatory inhibitors using a BV2 microglial cell model identified TMC 256 A1 (**10**) as the most potent candidate. Compound **10** significantly suppressed LPS-induced inflammation in BV2 cells without cytotoxicity. It concurrently inhibited LPS-triggered ROS overproduction and neutrophilic infiltration in zebrafish. Subsequent proteomics revealed that **10** targets NOS2 to modulate Alzheimer’s disease (AD)-associated pathways and the KEAP1-NRF2 axis. Molecular docking and dynamics simulations demonstrated that **10** occupies the NOS2 heme-binding pocket, thereby preventing dimerization and inhibiting enzymatic activity. Finally, **10** ameliorated locomotor deficits in an AD zebrafish model. Collectively, these findings highlight compound **10** as a candidate compound for preventing inflammatory and oxidative stress damage during treatment of neurodegenerative diseases, particularly AD.

## 1. Introduction

Neurodegenerative diseases represent a major class of neurological disorders characterized by diverse clinical and pathological features that affect specific neuronal subpopulations within distinct regions of the central nervous system. Although these disorders exhibit heterogeneous pathogenesis, such as disparate protein aggregations and genetic variations, they all share the common hallmark of chronic neuroinflammation [1]. Microglia-mediated neuroinflammation may play a central role in the progression of neurodegenerative disorders [2]. Alzheimer’s disease (AD), an irreversible and progressive neurodegenerative disorder, affects over 55 million people globally [3]. Currently, no reliable curative or preventative therapies exist for AD. This critical therapeutic gap underscores the urgency for developing effective AD treatments.

Central to neuroinflammatory processes, nitric oxide (NO) is a critical signaling molecule synthesized from L-arginine by nitric oxide synthase (NOS) [4]. Among NOS isoforms, inducible NOS (NOS2) is distinguished by its inflammation-responsive expression and sustained high-output NO production (>1 μM for days) [5]. In activated microglia, such prolonged NOS2-derived NO fluxes profoundly amplify neuroinflammatory cascades, thereby accelerating neurodegenerative pathology [6]. Given this mechanistic role, NOS2 inhibitors hold broad therapeutic potential in NOS2-mediated pathologies.

Recent years have witnessed significant traction in marine-derived fungi as pivotal sources for discovering anti-neuroinflammatory agents [7]. Numerous new secondary metabolites exhibiting potent anti-inflammatory activities have been identified from marine-derived fungal strains [8]. These compounds demonstrate not only favorable pharmacological profiles but also consistently low cytotoxicity and enhanced bioavailability, positioning them as promising drug leads for neurodegenerative disease therapeutics [9].

**Figure 1:**
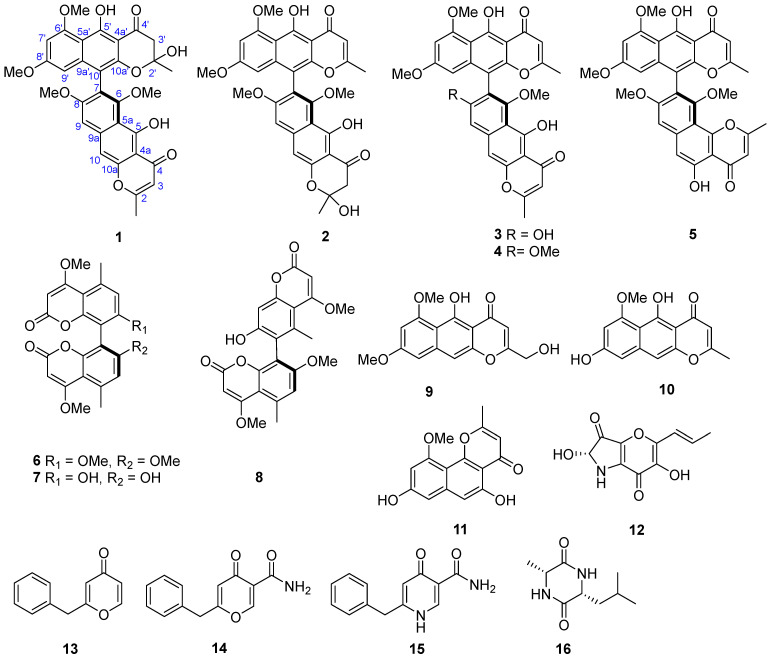
The chemical structures of compounds **1**–**16** isolated from *Aspergillus niger* 3A00562.

In this study, two new dimeric naphtho-*γ*-pyrone compounds (**1** and **2**) alongside 14 known compounds (**3**–**16**) were isolated from the deep-sea-derived fungus *Aspergillus niger* 3A00562 (Figure 1). Significantly, TMC 256 A1 (**10**) attenuated neuroinflammation by suppressing LPS-induced microglial activation. We detail the isolation, structural characterization, and bioactivity evaluation of these metabolites. Furthermore, network pharmacology and proteomics were integrated to elucidate the neuroprotective targets and mechanisms of **10**, with its efficacy subsequently validated in a zebrafish model. This work provides new molecular insights into the anti-inflammatory mechanism of **10** and its potential application in targeting NOS2 for neurodegenerative disease therapeutics.

## 2. Results

### 2.1. Chemical Constituents of Aspergillus niger and Structural Elucidation

By comparing the NMR and MS with the references, 14 known compounds were identified as (a*S*)-dianhydro-aurasperone C (**3**), (a*S*)-aurasperone A (**4**) [10], (a*S*)-fonsecinone A (**5**) [10], kotanin (**6**) [11], orlandin (**7**) [12], desertorin B (**8**) [13], nigerasperone A (**9**) [14], TMC 256 A1 (**10**) [15], TMC-256C1 (**11**) [16], pyranonigrin A (**12**) [17], aspergyllone (**13**) [18], carbonarone A (**14**) [19], aspernigrin A (**15**) [20], cyclo (Ala-Leu) (**16**) [21].

**Figure 2:**
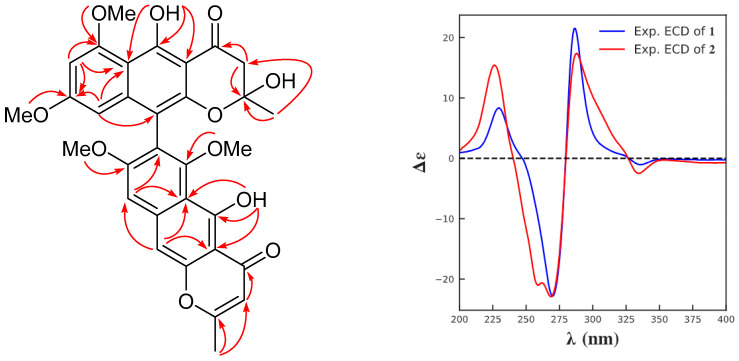
The key HMBC (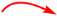) NMR correlations for compound **1** and experimental ECD spectra of **1** and **2**.

Compound **1** was isolated as a yellow powder. The molecular formula C_32_H_28_O_11_ was determined by the positive HRESIMS at *m*/*z* 611.1527 [M + Na]^+^ (calcd for C_32_H_28_O_11_Na, 611.1529), suggesting 19 degrees of unsaturation. The ^1^H NMR data of **1** (Table 1) presented signals for two methyls [*δ*_H_: 1.48 (3H, s, 2′-Me), 2.41 (3H, s, 2-Me)]; four methoxy [*δ*_H_: 3.42 (3H, s, 6-OMe), 3.62 (3H, s, 8′-OMe), 3.81 (3H, s, 8-OMe), 3.98 (3H, s, 6′-OMe)]; one methylene [*δ*_H_: 2.90 (1H, d, *J* = 17.2 Hz, H-3b′), 2.92 (1H, d, *J* = 16.8 Hz, H-3′a)]; four aromatic signals [*δ*_H_: 6.10 (1H, d, *J* = 2.3 Hz, H-7′), 6.34 (1H, d, *J* = 2.4 Hz, H-9′), 6.96 (1H, s, H-9), 7.14 (1H, s, H-10)], one olefinic proton [*δ*_H_: 6.05 (1H, s, H-3)], and two singlets due to hydrogen-bonded phenolic hydroxy groups [*δ*_H_: 14.56 (1H, s, 5′-OH), 14.89 (1H, s, 5-OH)]. The ^13^C NMR data showed 32 carbons consisting of two methyl carbons (*δ*_C_ 20.8, 28.4), four methoxy groups (*δ*_C_ 55.1, 55.9, 56.1, 61.8), one methylene carbon (*δ*_C_ 47.1), five sp^2^ methine (*δ*_C_ 96.0, 97.2, 101.3, 101.6, 107.3) and 20 non-protonated carbons (*δ*_C_ 100.2, 103.7, 104.7, 106.1, 107.5, 111.2, 118.8, 140.2, 142.6, 151.5, 153.1, 157.2, 160.3, 161.5, 161.8, 162.0, 164.8, 167.8, 184.5, 197.7). The presence of two carbonyl signals at *δ*_C_ 197.7 and 184.5 indicated that **1** was an asymmetric dimer of two linear naphtho-*γ*-pyrone monomers, of which one was hydrated at C2′–C3′. The key HMBC correlations from H-2-Me to C-2/C-3, H-3 to C-4, H-10 to C-4a/C-5a/C-9, H-9 to C-5a/C-7, H-9′ to C-10′/C-8′/C-5a′, H-7′ to C-5a′/C-6′/C-9′, H-2′-Me to C-2′/C-3′, H-3′ to C-2′/C-4′, 5-OH to C-4a/C-5/C-5a, 5′-OH to C-4a′/C-5′/C-5a′ that allowed assignment of the planar structural skeleton (Figure 2). A comparison of the ^13^C NMR data with those of fonsecinone B [22] showed a large degree of similarity except at C-5, C-8, C-9, C-10, C-4′, C-5′, C-5a′, and C-8′ [(Δ*δ*_C_ +0.8, −2.6, −1.3, −0.8, +1, +5.1, −2.6, −2.5) (Δ*δ*_C_ = *δ*_1_–*δ*_ref._). Hence, compound **1** is a new naphtho-*γ*-pyrone derivative.

The absolute configuration of **1** was assigned by the CD excitation chirality method. Compounds of naphtho-*γ*-pyrone usually display axial chirality due to the high energy barrier for rotation of the bond linking the individual aromatic systems [23,24]. The CD spectrum (Figure 2) of **1** showed positive Cotton effects at 286 (Δ*ε* + 21.5) and 230 (Δ*ε* + 8.3) nm, and negative Cotton effect at 270 (Δ*ε* − 22.8) nm, suggesting the (a*S*) absolute configuration for **1** [22,24,25,26]. Therefore, the structure of compound **1** was determined and named as (a*S*)-fonsecinone B. Similar to reported 2,3-hydrated naphtho-*γ*-pyrones, the configurations at the chiral centers C-2′ for **1** remained unknown [14,22,26,27].

Compound **2** was obtained as a yellow powder. The molecular formula was established as C_32_H_28_O_11_ (19 degrees of unsaturation) based on HRESIMS analysis, which showed a sodium adduct ion at *m*/*z* 611.1519 [M + Na]^+^ (calcd for C_32_H_28_O_11_Na, 611.1529). The high similarity between the 1D NMR spectra (Table 1) of compound **2** and fonsecinone D indicated that they share the same planar structure [28]. The absolute configuration of **2** was also deduced via the CD data. The CD spectrum of **2** (Figure 2) is nearly identical to that of **1**, indicating that **2** possesses the same absolute configuration as **1**. Therefore, the structure of **2** was elucidated and named (a*S*)-fonsecinone D.

### 2.2. Screening of Compounds for the Ability to Inhibit LPS-Induced Microglial Neuroinflammation

**Figure 3:**
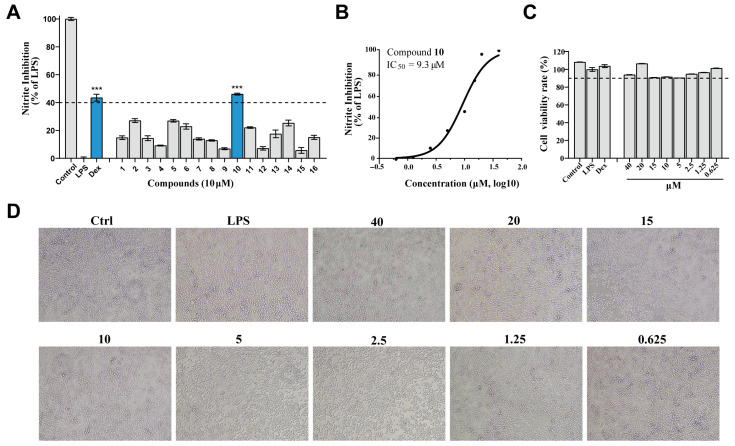
Identification of neuroprotective compounds through their anti-inflammatory properties. (**A**) Evaluation of the anti-inflammatory potential of marine-derived compounds **1**–**16** in BV2 cells, highlighting the inhibitory effects on nitric oxide (NO) production. (**B**) Determination of IC_50_ values for TMC 256 A1 (**10**), demonstrating its potency in inhibiting NO production, with **10** showing an IC_50_ of 9.3 μM. (**C**) Analysis of the cytotoxicity profile of **10** at various concentrations, indicating no significant cytotoxicity within the tested range. (**D**) Visualization of the cytotoxicity of **10**. Data were expressed as mean ± SEM. *** *p* < 0.001 compared with the LPS group.

To evaluate the neuroprotective potential of compounds **1**–**16**, their effects were investigated using the BV2 microglial cell line, a well-established model for screening inhibitors of neuroinflammation. As shown in Figure 3A, TMC 256 A1 (**10**) emerged as a promising inhibitor, exhibiting significant suppression at a concentration of 10 μM. The potency of compound **10** was further quantified, revealing an IC_50_ value of 9.3 μM (Figure 3B). Furthermore, to assess potential cytotoxicity, compound **10** was evaluated across the concentration range used in this study using the CCK-8 assay. Importantly, compound **10** showed no cytotoxic effects at the concentrations tested, as indicated by the results presented (Figure 3C,D).

### 2.3. TMC 256 A1 (**10**) Rescued LPS-Induced Inflammatory Impairment in Zebrafish Larvae

**Figure 4:**
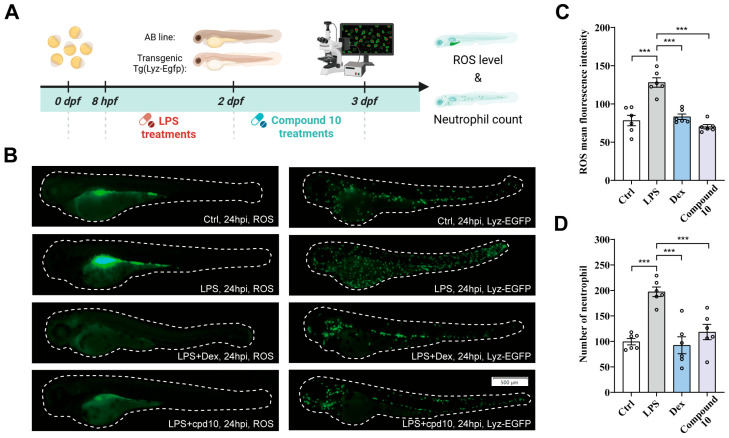
TMC 256 A1 (**10**) attenuates LPS-driven inflammatory impairment in zebrafish. (**A**) Timeline of zebrafish (wild-type AB strain and transgenic Tg(Lyz-Egfp)) maintenance and treatment with LPS (50 µg/mL), Dex (10 μM) and **10** (10 μM). (**B**) Fluorescence images of ROS (using DCFH-DA) and neutrophil in the LPS-induced in vivo zebrafish system. (**C**,**D**) Quantification of ROS fluorescent intensity and Lyz:Egfp cells in each group (*n* = 6). Data were expressed as mean ± SEM. *** *p* < 0.001 compared with the LPS group.

Based on the previous neuroprotective effects observed in vitro for compound **10**, we employed both wild-type AB strain and transgenic zebrafish models to evaluate its in vivo therapeutic efficacy following LPS challenge (Figure 4A). Zebrafish are a well-suited model for assessing neuroprotection due to their practical and cost-effective experimental benefits. Consistent with neuroinflammatory pathology, LPS exposure significantly elevated intracellular reactive oxygen species (ROS) levels in larval zebrafish (Figure 4B,C). This LPS-induced oxidative stress was effectively mitigated by treatment with both dexamethasone (Dex) and compound **10**. As a physiological hallmark of activated inflammatory immunity, a significant increase in neutrophil accumulation was observed upon LPS stimulation (Figure 4B,D). Compound **10** administration substantially reversed this pro-inflammatory neutrophil response. Collectively, these results demonstrate the therapeutic potential of compound **10** in mitigating key pathological features of inflammation in vivo.

### 2.4. Quantitative Proteomics Analysis and Predicting NOS2 as a Key Target

**Figure 5:**
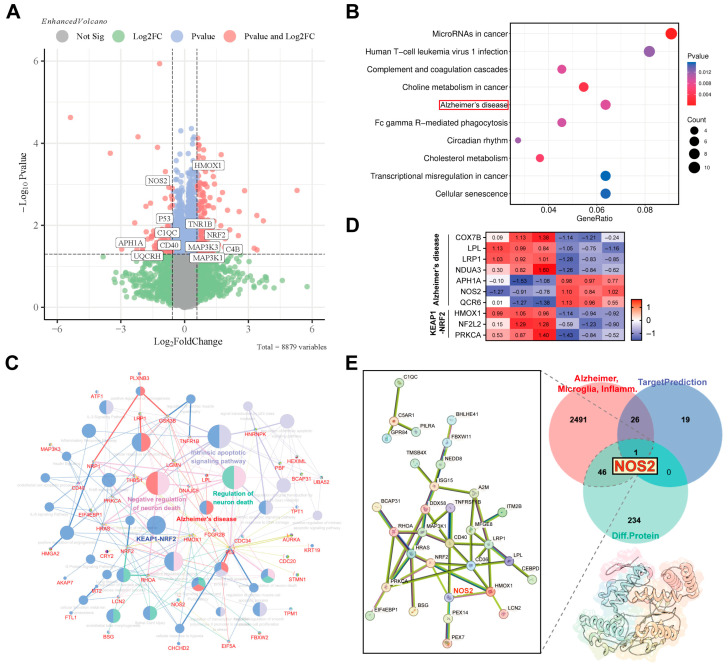
Quantitative proteomics analysis and key target prediction. (**A**) Volcanogram representation of differential expression proteins after TMC 256 A1 (**10**) treatments relative to LPS-induced group in BV2 cell. (**B**) Bubble chart of the top 10 enriched pathways. (**C**) Pathway–protein interaction network constructed to visualize connections between multi-cluster functional pathways and proteins. (**D**) Secondary classification annotation of KEGG. Heatmap of significant differential protein abundances about Alzheimer’s disease and the KEAP1-NRF2 axis. (**E**) PPI network of targets with more than two and three intersections in the multivariate Venn diagram.

To elucidate the pharmacological basis underlying the neuroprotective effects of compound **10**, quantitative proteomic analysis was performed on BV2 cells treated with LPS alone or compound **10** + LPS. Comparative analysis revealed 193 significantly upregulated proteins and 90 significantly downregulated proteins in the **10** + LPS group relative to the LPS control group (*p* < 0.05, |Log_2_FC| > 0.5; Figure 5A). Notably, KEGG pathway enrichment analysis indicated significant enrichment within the Alzheimer’s disease signaling pathway among the differentially expressed proteins (Figure 5B). Subsequent multi-cluster functional analysis using ClueGO further confirmed enrichment of differentially abundant proteins in pathways associated with both Alzheimer’s disease and the KEAP1-NRF2 axis (Figure 5C), supported by a corresponding differential heatmap of relevant proteins (Figure 5D). To identify potential direct targets mediating **10**’s anti-inflammatory action, we utilized the SwissTargetPrediction database to predict compound 10’s binding targets. These predicted targets were then intersected with 2040 proteins (retrieved from the GeneCards database) linked to microglial activation, inflammation, and Alzheimer’s disease pathogenesis. A comparative analysis of this intersection set with the proteomic targets identified by mass spectrometry was performed using jvenn. This integration analysis pinpointed NOS2 (inducible nitric oxide synthase) as a high-confidence candidate central to the overlap and the most plausible direct binding target for compound **10** in the context of Alzheimer’s disease therapy (Figure 5E). Collectively, these findings strongly suggest that compound **10** protects against LPS-induced neuroinflammation by modulating key signaling cascades, including the KEAP1-NRF2 pathway, likely through direct targeting of NOS2.

### 2.5. Binding Analysis of TMC 256 A1 (**10**) with NOS2

**Figure 6:**
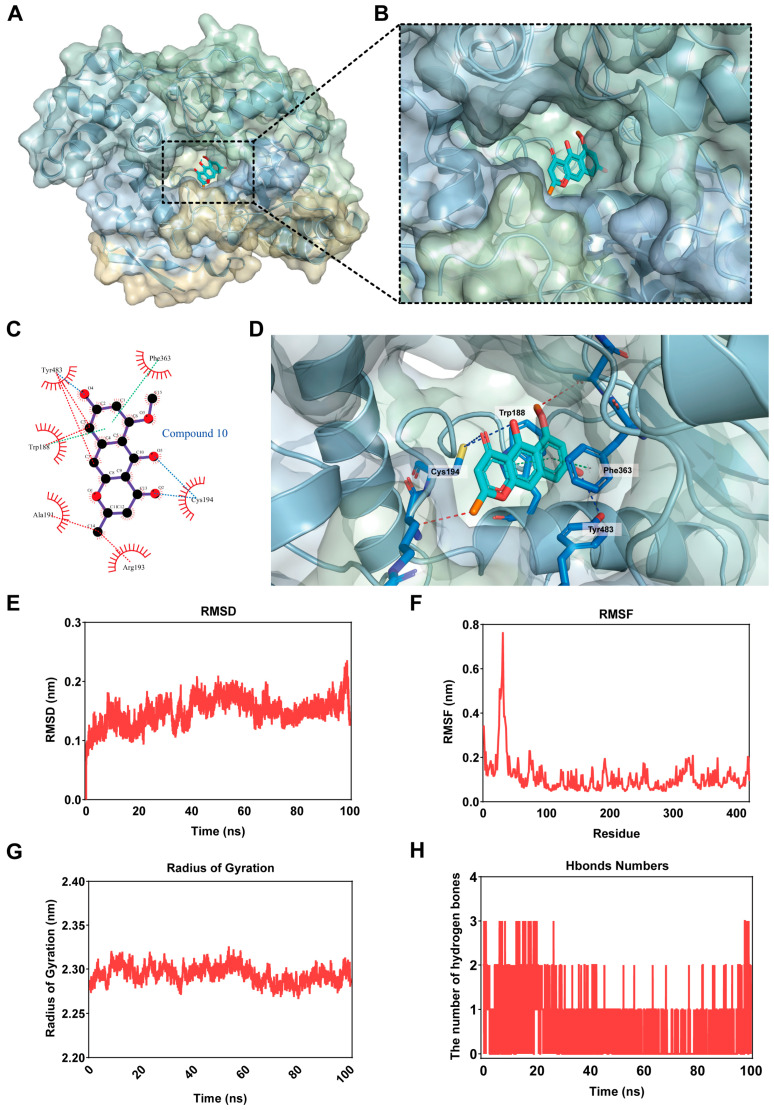
Molecular docking configuration of TMC 256 A1 (**10**) to the NOS2. (**A**) Molecular docking results of **10** and NOS2, the total view. (**B**) Side view of symmetrical dimer of the complex. (**C**) The detailed interaction view of NOS2 pertinent amino acids residues with **10**; hydrogen bonds: blue lines; hydrophobic interactions: red lines; π–alkyl interactions: green lines. (**D**) Compound **10** binding sites in the crystal structure. (**E**) The RMSD values of **10**-NOS2 complexes. (**F**) The RMSF values of **10**-NOS2 complexes. (**G**) The Rg values of **10**-NOS2 complexes. (**H**) The hydrogen (**H**) bond numbers in **10**-NOS2 complexes.

Here, molecular docking was used to investigate the interaction between **10** and NOS2 (PDB: 4JS9) and its binding mode. The optimal binding configuration was identified, characterized by high binding affinity and a favorable binding free energy of −9.5 kcal/mol (Figure 6A,B). From the possible sites (Figure 6C), **10** was in a hydrophobic pocket formed by amino acid residues such as tyrosine (Tyr) 483, phenylalanine (Phe) 363, cysteine (Cys) 194, arginine (Arg) 193, alanine (Ala) 191 and tryptophan (Trp) 188 (Figure 6B). The docking results showed the existence of conventional hydrogen bonds, hydrophobic interactions and π–alkyl interactions between NOS2-related amino acid residues and **10**, among which hydrogen bonds were the most important. Specifically, two hydrogen bonds were formed between the keto and hydroxyl groups of **10** and the amino acid residues Tyr 483 and Cys 194 of the NOS2 protein (Figure 6D). These hydrogen bonds exhibited strong interactions, with donor–acceptor distances of 2.7 and 3.5 Å. Additionally, **10** established π-π stacking with Trp 188, and Phe 363, while being stabilized within the hydrophobic cavity of the NOS2 protein through interactions with surrounding hydrophobic and hydrophilic residues.

The root-mean-square deviation (RMSD) of 10- NOS2 complex fluctuated within 0.1 nm (Figure 6E), with root-mean-square fluctuation (RMSF) values of most residues in the binding pocket below 0.2 nm (Figure 6F). The radius of gyration (Rg) remained stable at ~2.3 nm (Figure 6G), collectively demonstrating stable and compact complex conformation. The number of hydrogen bonds between the 10-NOS2 complex system ranged from 0 to 3, and in most cases, the complex has approximately one or two hydrogen bonds (Figure 6H), implying persistent polar interactions stabilizing the complex.

### 2.6. Dyskinesia Rehabilitation Effects of TMC 256 A1 (***10***) in AlCl_3_-Induced AD Zebrafish Larvae

**Figure 7:**
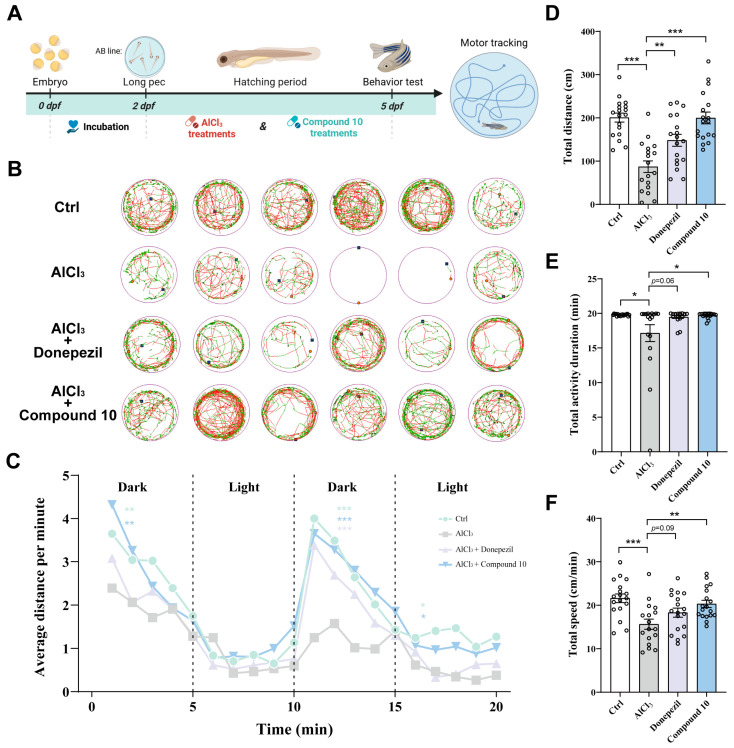
TMC 256 A1 (**10**) attenuates AlCl_3_-driven behavioral impairment in zebrafish. (**A**) Timeline of zebrafish (wild-type AB strain) maintenance and treatment with AlCl_3_ (100 μM), Donepezil (4 μM) and **10** (10 μM). (**B**) The representative trajectory of zebrafish larvae was recorded for 20 min. (**C**) Locomotor patterns and average swimming distance during dark–light stimulations of zebrafish after exposure to AlCl_3_ and drug treatment from 2 to 5 dpf. (**D**–**F**) The total swimming distance, duration and average swimming velocity of each group were measured for 20 min on day 5 (*n* = 18, per group). Data were represented as mean ± SEM. * *p* < 0.05, ** *p* < 0.01, and *** *p* < 0.001 compared with the AlCl_3_ group.

Behavioral assessments were conducted on zebrafish larvae at 5 days post-fertilization (5 dpf; Figure 7A). Consistent with prior findings, aluminum chloride (AlCl_3_) exposure induced a marked reduction in larval locomotor activity (Figure 7B) [29]. This result confirms the successful induction of Alzheimer’s disease (AD)-like pathology in the zebrafish model. Significantly, treatment of AD-model zebrafish with either donepezil (positive control) or compound **10** alleviated the AlCl_3_-induced locomotor deficits. Specifically, both compounds increased the total distance moved, total active time, and swimming velocity compared to the untreated AD model group (Figure 7D–F). Zebrafish larvae exhibit distinct and quantifiable swimming patterns in response to light–dark transitions, a behavior intrinsically linked to social interaction, anxiety levels, learning/memory function, and defense mechanisms [30]. Notably, under dark-phase conditions, zebrafish treated with either donepezil or compound **10** demonstrated significantly greater distances traveled than those in the AD model group (Figure 7C). Collectively, these results strongly suggest that compound **10** enhanced motor performance and exerted protective effects against AlCl_3_-induced AD-like behavioral symptoms in zebrafish.

## 3. Discussion

In this study, 16 compounds were isolated from the deep-sea-derived fungus *Aspergillus niger* 3A00562, including five dimeric naphtho-*γ*-pyrone (**1**–**5**), three bicoumarins (**6**–**7**). The absolute configuration of dimeric naphtho-*γ*-pyrones was dominated by the chiral axes between the two naphthopyranone moieties (atropisomerism). The absolute configurations of dimeric naphtho-*γ*-pyrones have been determined by circular dichroism (CD). According to the literature [10,24], (a*S*)-configured dimeric naphtho-*γ*-pyrones exhibit a first positive Cotton effect in the long-wavelength region, a negative Cotton effect at middle wavelength and then a positive Cotton effect at shorter one. After the 1D NMR and MS data of **1**–**5** were compared with literature data, it was revealed that these compounds possessed the same planar structures as those of fonsecinone B (**1**) [22], fonsecinone D (**2**) [28], dianhydro-aurasperone C (**3**) [10], aurasperone A (**4**) [10], fonsecinone A (**5**) [10], respectively; The experimental CD spectra of compounds **1**–**5** displayed Cotton effects similar to those of (a*S*)-configured analogs (Figure S42), which indicated that the configuration of compounds **1**–**5** were a*S*, among them, (a*S*)-dianhydro-aurasperone C (**3**), (a*S*)-aurasperone A (**4**), and (a*S*)-fonsecinone A (**5**) have been reported in previous papers [10], and compounds **1** and **2** were reported for the first time in this article, namely, (a*S*)-fonsecinone B (**1**) and (a*S*)-fonsecinone D (**2**), respectively. Further analysis of compound **1** using a chiral column confirmed that compounds **1**–**5** were all isolated as the a*S* configuration (Figure S47). In addition, the structures of the known compounds **6**–**7** were identified by comparison of their spectroscopic data with those reported in the literature [31,32], all bicoumarins were optically active, and their absolute configuration was assigned as a*S*.

Alzheimer’s disease (AD) represents the most prevalent neurodegenerative disorder in clinical practice, yet its complex pathogenesis impedes the development of effective therapeutics. Accumulating evidence in recent years implicates neuroinflammation and oxidative stress in AD progression, wherein suppression of neuroinflammatory cascades has emerged as a viable therapeutic strategy. Among 16 natural products isolated from deep-sea-derived fungi, TMC 256 A1 (**10**) (a naphthopyrones-type production) was identified as possessing both anti-inflammatory and antioxidant activities. Although compound **10** was previously reported to inhibit IL-4 signaling transduction in macrophages [16], its biological activities remained largely unexplored. Here, we report for the first time that **10** mitigates AD symptoms by directly targeting NOS2 to attenuate NO production, thereby conferring coordinated anti-inflammatory and antioxidant effects.

In neurodegenerative contexts, accumulated evidence indicates that excessive reactive oxygen species (ROS) drive cellular damage and death. Notably, pathological overproduction of the inflammatory mediator nitric oxide (NO) can precipitate oxidative stress, mechanistically intersecting with ROS accumulation [33]. Our findings reveal that **10** significantly suppresses NO generation in LPS-induced neuroinflammation models and reduces ROS burden on zebrafish inflammation assays. This functional profile aligns with documented anti-inflammatory and antioxidant properties of marine natural products [34].These observations collectively suggest the potential of **10** as a neuroprotective agent with anti-inflammatory efficacy.

Integrating proteomics and network-based pharmacological analysis, we deciphered the anti-neuroinflammatory mechanism of compound **10**. Functional enrichment clustering revealed significant associations between differentially expressed proteins and key inflammatory pathways—including Alzheimer’s disease, KEAP1-NRF2 signaling, and regulation of neuronal apoptosis—suggesting compound **10** sustains neuronal survival through dual anti-inflammatory/antioxidant actions. Among 47 overlapping targets identified from multi-database mining and proteomics, protein–protein interaction (PPI) networks highlighted NOS2, LPL, LRP1, NRF2, and HMOX1 as potential neurofunctional modulators. Crucially, NOS2 was predicted as a direct target of compound **10**. Structurally, NOS2 monomers comprise a reductase domain and an oxygenase domain, the latter containing binding sites for L-arginine, heme, and tetrahydrobiopterin (H4B). Heme-dependent dimerization—requiring coordinated H4B, substrate, and heme binding within the oxygenase domain—is essential for fully coupled enzymatic activity [35,36]. Molecular docking corroborated NOS2’s role in regulating neuroinflammation, demonstrating that **10** binds tightly to the hydrophobic cavity of the oxygenase domain via hydrogen bonding and π-π stacking, thereby inhibiting heme-dependent dimer formation and suppressing catalytic function.

Zebrafish represent a superior in vivo system for human disease modeling and drug discovery, owing to their extensive genetic homology with humans, rapid development, and small size [37]. Aluminum chloride-induced zebrafish Alzheimer’s models recapitulate behavioral deficits, as documented in prior studies [29]. In this model, compound **10** significantly enhanced locomotor capacity. Moreover, during light/dark transition assays, treated zebrafish exhibited longer travel distances in dark phases than the disease group.

Collectively, this work demonstrates that **10** confers neuroprotection by targeting neuronal NOS2 to suppress NO and ROS production, consequently attenuating neuroinflammation and oxidative stress. The compound further modulates multiple signaling pathways, including Alzheimer’s disease-associated cascades and KEAP1-NRF2 axis regulation. These findings provide robust molecular insights into the anti-neuroinflammatory mechanism of **10**, highlighting its translational potential for neurodegenerative therapeutics.

## 4. Materials and Methods

### 4.1. General Experimental Procedures

Optical rotations were recorded on an Anton Paar MCP 100 polarimeter (Anton Paar Trading Co., Ltd., MCP100, Shanghai, China). UV spectra were recorded on a UV-8000 UV/Vis spectrometer (Shanghai Yuanxi Instrument Co, Ltd., Shanghai, China). CD spectra were recorded on Chirascan spectropolarimeter (Applied Photophysics, Leatherhead, United Kingdom). The NMR spectra were recorded on a Bruker AVANCE III 400 MHz spectrometer (Bruker, Fällanden, Switzerland). Chemical shifts were recorded in δ values using solvent signals (DMSO-*d*_6_: *δ*_H_ 2.50 and *δ*c 39.5; CD_3_OD: *δ*_H_ 3.31 and *δ*c 49.0; CDCl_3_: *δ*_H_ 7.26 and *δ*c 77.0) as references. The HRESIMS spectra was recorded on Thermo Q-Exactive Focus tandem mass spectrometry (Thermo Scientific, Waltham, MA, USA). The preparative and semipreparative HPLC were performed on an Agilent Technologies 1260 infinity instrument equipped with the DAD detector (Agilent Technologies, San Diego, CA, USA). Column chromatography (CC) was performed on silica gel, ODS, and Sephadex LH-20. The TLC plates were visualized under UV light or by spraying with 10% H_2_SO_4_.

### 4.2. Fungal Material

The fungal strain *Aspergillus niger* 3A00562 was isolated from deep-sea sediments of the South Atlantic at a depth of −3059 m. It was identified to be an *A. niger* (GenBank accession number KP003820), as the ITS DNA gene sequence alignment demonstrated that it was similar to *Aspergillus niger* ATCC 16888 (GenBank accession number NR_111348.1, similarity 100.00%). The voucher strain was preserved at the Hainan Medical University, Haikou, China, and given the accession number MCCC 3A00562.

### 4.3. Fermentation, Extraction, and Isolation

*Aspergillus niger* 3A00562 was cultured on a PDA plate at 25 °C for 3 days. Then, the fresh mycelia were inoculated into 50 × 1 L Erlenmeyer flasks liquid medium with each 1 L tap water (pH 7.5) containing mannitol (20 g), monosodium glutamate (5 g), maltose (3 g), yeast extract (3 g), glucose (10 g), and soya peptone (5 g). The flasks were statically incubated at 25 °C for 47 days. Following this, the fermentation broth was extracted with EtOAc three times to provide a crude extract. The extract was redissolved in MeOH and extracted with petroleum ether (PE) three times. The MeOH solution was evaporated under reduced pressure to obtain a defatted extract (52 g), which was subjected to CC over silica gel using sequential gradient elution with CH_2_Cl_2_−MeOH (100%→90%) to obtain three fractions (Fr.1–Fr.3) based on TLC properties. Fraction Fr.1 (6.8 g) was separated into three subfractions (Fr.1-1–Fr.1-3) by CC over ODS with MeOH-H_2_O (50%→100%). Subfraction Fr.1-1 (208.2 mg) was separated by CC on Sephadex LH-20 (MeOH) to give **14** (15.4 mg). Further purification using preparative TLC (CH_2_Cl_2_−MeOH, 20:1) provided **13** (79 mg). Subfraction Fr.1-2 (1400 mg) was subjected to CC on Sephadex LH-20 (MeOH), followed by purification using HPLC (MeOH-H_2_O, 75%→100%) to afford **1** (8 mg, *t*_R_ = 22 min), **2** (7 mg, *t*_R_ = 19 min), **3** (2 mg, *t*_R_ = 20 min), **4** (7 mg, *t*_R_ = 26.5 min) and **5** (9 mg, *t*_R_ = 24.5 min). Subfraction Fr.1-3 (146 mg) was subjected to CC on Sephadex LH-20 (MeOH) and preparative TLC (CH_2_Cl_2_-MeOH, 30:1) to yield **6** (4 mg). Final purification using preparative HPLC (MeOH-H_2_O, 55%) provided **10** (4.3 mg, *t*_R_ = 26 min) and **11** (3.4 mg, *t*_R_ = 30 min). Fraction Fr.2 (8.3 g) was chromatographed over ODS using gradient elution of MeOH-H_2_O (5%→100%) to get five subfractions (Fr.2-1–Fr.2-5). Subfractions Fr.2-1 (301.8 mg) and Fr.2-3 (730 mg) were subjected to CC on Sephadex LH-20 (MeOH), followed by purification using HPLC (MeOH-H_2_O, 30%→60%) to afford **7** (7 mg, *t*_R_ = 15 min) and **9** (2.4 mg, *t*_R_ = 17.5 min), respectively. Subfraction Fr.2-2 (786.9 mg) was separated subsequently by CC over Sephadex LH-20 (MeOH) and crystallization (MeOH) to yield **8** (20 mg). Subfraction Fr.2-4 (477 mg) and Fr.2-5 (600 mg) were separated by CC on Sephadex LH-20 (MeOH) to give **15** (13 mg) and **16** (16 mg). Fraction Fr.3 (8.8 g) was subjected to CC over ODS with MeOH-H_2_O (10%→60%), followed by purification using CC on Sephadex LH-20 (MeOH) and HPLC (MeOH-H_2_O, 30%→60%) to yield **12** (46 mg, *t*_R_ = 18.6 min).

(a*S*)-Fonsecinone B (**1**): Yellow powder; ${\left[\alpha \right]}_{D}^{25}$ −12 (c 0.10, MeOH); UV (MeOH) *λ*_max_ (log *ε*) 281 (2.52), 242 (2.11), 227 (2.22) nm; ECD (MeOH) *λ*_max_ (Δ*ε*) 286 (+7.66), 270 (−8.14), 230 (+2.97) nm; ^1^H and ^13^C NMR data, Table 1; HRESIMS *m*/*z* 611.1527 [M + Na]+ (calcd for C_32_H_28_O_11_Na, 611.1529).

(a*S*)-Fonsecinone D (**2**): Yellow powder; ${\left[\alpha \right]}_{D}^{25}$ −41 (c 0.10, MeOH); UV (MeOH) *λ*_max_ (log *ε*) 280 (2.97), 260 (2.74), 235 (2.90) nm; ECD (MeOH) *λ*_max_ (Δ*ε*) 288 (+6.21), 269 (−8.18), 226 (+5.51) nm; ^1^H and ^13^C NMR data, Table 1; HRESIMS *m*/*z* 611.1527 [M + Na]^+^ (calcd for C_32_H_28_O_11_Na, 611.1529).

### 4.4. Materials

Dulbecco’s Modified Eagle Medium (DMEM), trypsin, and antibiotics were obtained from Basal Media Technologies (Shanghai, China, L120KJ). Fetal bovine serum and Griess Reagent Kit were obtained from Thermo Fisher Scientific, Inc. (Shanghai, China, A5669701). Lipopolysaccharide (LPS) was obtained from Sigma-Aldrich, Inc. (Shanghai, China, L4391). Dexamethasone (D129705) and Donepezil (D129948) were supplied by Aladdin Biochemical Technology Co., Ltd. (Shanghai, China). The Cell Counting Kit-8 (CCK8) was purchased from Topscience Biotechnology Co., Ltd. (Shanghai, China, C0005). DCFH-DA (R252) was supplied by Dojindo Chemical Technology Co., Ltd. (Dojindo, Shanghai, China).

### 4.5. Cell and Animals

The murine microglial cell line BV2 was derived from the American Type Culture Collection (Rockville, MD, USA). BV2 cell was cultured in DMEM, supplemented with 10% FBS and 1% antibiotics, at 37 °C in a humidified incubator with 5% CO_2_. Cells were passed 30–40 times or replaced from the original frozen stock every 3 months.

Wild-type (AB strain) and transgenic (Tg: Lyz-Egfp) zebrafish embryos were obtained from the Zebrafish Experimental Platform of Public Research Center at Hainan Medical University (Haikou, China) and collected in 100 mm dishes. Then, the zebrafish embryos were maintained under a standard temperature at 28 ± 0.5 °C with a 14 h light/10 h dark cycle. All experiments were conducted in accordance with the Association for Assessment and Accreditation of Laboratory Animal Care International (AAALAC, 001458) and Institutional Animal Care and Use Committee (IACUC-2023–6463-01) guidelines.

### 4.6. Anti-Inflammatory Activity

BV-2 cell culture and compound treatment were performed as previously reported [34]. Cells were seeded on a 24-well plate at a density of 1.5 × 10^5^ cells per well and allowed to adhere overnight. The following day, cells were treated with fresh medium containing the tested compounds at specified concentrations for 1 h, followed by exposure to LPS (1 µg/mL). Control groups were treated with a 0.1% DMSO solution. The concentration of nitrite present in the culture medium was assessed using the Griess Reagent Kit. Following the manufacturer’s instructions, the absorbance at a wavelength of 560 nm was recorded using the Infinite 200Pro microplate reader (TECAN, Männedorf, Switzerland).

### 4.7. Cytotoxicity Test

BV2 cells were seeded in 96-well plates at a density of 5 × 10^3^ cells per well with 200 μL of culture medium containing 10% FBS and incubated for 24 h. Following this, the cells were treated with various concentrations of the test compounds in 100 μL of 10% FBS culture medium for 48 h. Cell viability was then assessed using the CCK8 kit. In accordance with the manufacturer’s instructions for the reagent, the optical density (OD) at 450 nm was measured using the Infinite 200Pro microplate reader (TECAN, Männedorf, Switzerland).

### 4.8. Establishment and Treatment of Inflammatory Zebrafish Model

At 8 hpf, AB strain and transgenic Tg(Lyz: Egfp) larvae were exposed to LPS (50 µg/mL) as previously described [38]. After 2 days, the zebrafish were treated with Dex (10 μM) and 10 (10 μM). After 24 h, in vivo ROS levels were determined using DCFH-DA and counted neutrophils numbers. Fluorescently labeled neutrophils were imaged and counted using a MVX10 stereo fluorescence microscope (Olympus Corporation, Tokyo, Japan).

### 4.9. Proteomic Analyses

Cell pellets were resuspended in 0.5% sodium deoxycholate, incubated at 95 °C for 5 min in a metal bath, followed by ice-cold sonication. After centrifugation at 12,000× *g* (20 min, 4 °C), supernatants underwent BCA quantification; 30 µg protein aliquots were reduced/alkylated with 20 µL 10 mM DTT/20 mM CAA (95 °C, 1000 rpm, 5 min). After 10 min cooling, tryptic digestion proceeded at 37 °C (1000 rpm, 2 h, 1:50 enzyme-to-protein ratio), terminated by 55 µL 0.1% TFA. Samples were desalted via solid-phase extraction: loaded onto microcolumns, washed twice with 100 µL 0.1% TFA under positive pressure, and eluted twice with 50 µL 50% ACN/0.1% TFA. Eluents were concentrated by vacuum centrifugation.

Chromatographic separation used a Vanquish UHPLC system with ReproSil C18 (Dr. Maisch HPLC GmbH, Ammerbuch, Germany) column (18 cm) at 300 nL/min, mobile phase A (0.1% formic acid in water) and B (0.1% formic acid in 80% ACN) with gradient: 5–35% B over 60 min. MS analysis employed a Thermo Orbitrap Eclipse in DIA mode: MS1 scans at 400–800 *m*/*z* (120K resolution), MS2 with 45 consecutive 9-Da isolation windows (60K resolution) across 400–800 *m*/*z*.

### 4.10. Target Screening of Compound and Diseases

SwissTargetPrediction “http://swisstargetprediction.ch/ (accessed on 12 October 2025)” and GeneCard “https://www.genecards.org/ (accessed on 12 October 2025)” were used to predict the relevant targets and disease of compound 10. The compound potential targets were used to search for and identify intersections with previously differentially expressed proteins to identify the associated targets for compound and neurodegenerative disease, a Venn diagram was used. Cytoscape 3.9.1 was applied to construct the protein target network of compound 10. The key targets and pathways were analyzed for enrichment in GO and KEGG.

### 4.11. Molecular Docking

The structure of the protein (NOS2: 4JS9) was downloaded from the Protein Data Bank (PDB). The 3D structure of the small molecule was constructed from yinfotek computing platform “https://cloud.yinfotek.com (accessed on 12 October 2025)” and energy minimization was carried out under the MMFF94 force field. AutoDock Vina 1.1.21 software was adopted for molecular docking and PyMol 2.4.0 (The Scripps Research Institute, La Jolla, CA, USA) was used to remove water molecules, salt ions, and small molecules from the protein. The docking box was then set up to encase the entire protein structure. The Lamarckian Genetic Algorithm (LGA) was employed to conduct a conformational search. For docking, all parameters were kept at default. The docking scores (reported in kcal/mol) reflect binding energy, where more negative values indicate stronger binding affinity. The output docked conformation of the proteins was considered as the definitive conformation. LigPlot + 2.3.1 (EMBL-EBI) was utilized to analyze the interaction forces, and finally, PyMol 2.4.0 (DeLano Scientific LLC., South San Francisco, CA, USA) was used for visualization.

### 4.12. Dynamics Simulations

The 10-NOS2 complex obtained based on docking was used as the initial structure for all-atom molecular dynamics simulations, which were performed using AMBER 20 software. Computational modeling and dynamic simulations were implemented on the yinfotek computing platform.

### 4.13. Zebrafish Behavioral Test

The 5 dpf zebrafish larvae were transferred into new 24-well plates at a density of 1/well, which was allowed to accommodate the new environment for 20 min. Then, the larval movements were recorded for at least 20 min. The videos were analyzed by ZebraLab software 5.15 (Viewpoint Life Sciences, Chassieu, France) with the total moving distance and average moving speed. Meanwhile, the trajectory was also recorded.

### 4.14. Statistical Analysis

Data were expressed as mean ± standard error of the mean and were analyzed by one-way ANOVA (Turkey test) using Prism 8.0 (GraphPad, La Jolla, CA, USA). Statistical significance was set at * *p* < 0.05, ** *p* < 0.01, and *** *p* < 0.001.

## Figures and Tables

**Table 1:** ^1^H (400 Hz) and ^13^C (100 Hz) NMR spectroscopic data of compounds **1** and **2** in CDCl_3_.

NO.	1		2	
	*δ*_C_, Type	*δ*_H_ (*J* in Hz)	*δ*_C_, Type	*δ*_H_ (*J* in Hz)
2	167.8, C		100.1, C	
3	107.3, CH	6.05 s	47.2, CH_2_	3.00 s
4	184.5, C		196.6, C	
4a	104.7, C		103.5, C	
5	162.0, C		164.0, C	
5a	111.2, C		110.7, C	
6	157.2, C		159.4, C	
7	118.8, C		116.8, C	
8	160.3, C		161.0, C	
9	101.6, CH	6.96 s	102.7, CH	6.84 s
9a	140.2, C		142.7, C	
10	101.3, CH	7.14 s	101.8, CH	6.70 s
10a	153.1, C		153.3, C	
2′	100.2, C		167.6, C	
3′	47.1, CH_2_	2.91 (dd, 21.2, 11.4)	107.2, CH	5.97 s
4′	197.7, C		184.6, C	
4′a	103.7, C		104.2, C	
5′	164.8, C		162.6, C	
5′a	106.1, C		108.5, C	
6′	161.5, C		160.9, C	
7′	97.2, CH	6.10 s	96.9, CH	6.41 s
8′	161.8, C		161.4, C	
9′	96.0, CH	6.34 s	96.4, CH	6.21 s
9′a	142.6, C		140.7, C	
10′	107.5, C		105.1, C	
10′a	151.5, C		150.8, C	
2-Me	20.8, CH_3_	2.41 s	28.7, CH_3_	1.80 s
2′-Me	28.4, CH_3_	1.48 s	20.7, CH_3_	2.12 s
6-OMe	61.8, CH_3_	3.42 s	61.9, CH_3_	3.42 s
6′-OMe	56.1, CH_3_	3.98 s	56.2, CH_3_	4.01 s
8-OMe	55.9, CH_3_	3.81 s	55.9, CH_3_	3.75 s
8′-OMe	55.1, CH_3_	3.62 s	55.2, CH_3_	3.62 s
5-OH		14.56 s		14.14 s
5′-OH		14.89 s		15.30 s

## Data Availability

The raw data supporting the conclusions of this article will be made available by the authors on request.

## Supplementary Materials

The following supporting information can be downloaded at: https://www.mdpi.com/article/10.3390/md24040125/s1, Figures S1–S45: The NMR, HRESIMS, Optical rotation spectra of compounds **1**–**16**; Figure S46: Experimental ECD spectra of compounds **1**–**5**; Figure S47: HPLC chromatogram of compound **1** analyzed using an OptiChiral A1 column.
