# Supplementary material for: Anti-Neuroinflammatory Naphtho-γ-Pyrones from a Deep-Sea-Derived Fungus Aspergillus niger 3A00562
Source: Mar Drugs. 2026 Mar 27;24(4):125. doi: 10.3390/md24040125 (PMC13117293; doi:10.3390/md24040125)
Supplement: Supplementary file 1 [file marinedrugs-24-00125-s001.zip › marinedrugs-4153702-supplementary.pdf]

Supplementary Material

Content

Figure S1. ^{1}H NMR spectrum of compound **1** in $CDCl_3$.
Figure S2. ^{13}C NMR spectrum of compound **1** in $CDCl_3$.
Figure S3. HSQC spectrum of compound **1** in $CDCl_3$.
Figure S4. ^{1}H-^{1}H COSY spectrum of compound **1** in $CDCl_3$.
Figure S5. HMBC spectrum of compound **1** in $CDCl_3$.
Figure S6. NOESY spectrum of compound **1** in $CDCl_3$.
Figure S7. The positive HR-ESI-MS spectrum of compound **1**.
Figure S8. Optical rotation spectrum of compound **1**.
Figure S9. ^{1}H NMR spectrum of compound **2** in $CDCl_3$.
Figure S10. ^{13}C NMR spectrum of compound **2** in $CDCl_3$.
Figure S11. HSQC spectrum of compound **2** in $CDCl_3$.
Figure S12. ^{1}H-^{1}H COSY spectrum of compound **2** in $CDCl_3$.
Figure S13. HMBC spectrum of compound **2** in $CDCl_3$.
Figure S14. NOESY spectrum of compound **2** in $CDCl_3$.
Figure S15. The positive HR-ESI-MS spectrum of compound **2**.
Figure S16. Optical rotation spectrum of compound **2**.
Figure S17. ^{1}H NMR spectrum of compound **3** in $CDCl_3$.
Figure S18. ^{13}C NMR spectrum of compound **3** in $CDCl_3$.
Figure S19. ^{1}H NMR spectrum of compound **4** in $CDCl_3$.
Figure S20. ^{13}C NMR spectrum of compound **4** in $CDCl_3$.
Figure S21. ^{1}H NMR spectrum of compound **5** in $CDCl_3$.
Figure S22. ^{13}C NMR spectrum of compound **5** in $CDCl_3$.
Figure S23. ^{1}H NMR spectrum of compound **6** in $CDCl_3$.
Figure S24. ^{13}C NMR spectrum of compound **6** in $CDCl_3$.
Figure S25. ^{1}H NMR spectrum of compound **7** in MeOD-d_4.
Figure S26. ^{13}C NMR spectrum of compound **7** in MeOD-d_4.
Figure S27. ^{1}H NMR spectrum of compound **8** in DMSO-d_6.
Figure S28. ^{13}C NMR spectrum of compound **8** in DMSO-d_6.
Figure S29. ^{1}H NMR spectrum of compound **9** in DMSO-d_6.
Figure S30. ^{13}C NMR spectrum of compound **9** in DMSO-d_6.
Figure S31. ^{1}H NMR spectrum of compound **10** in DMSO-d_6.
Figure S32. ^{13}C NMR spectrum of compound **10** in DMSO-d_6.
Figure S33. The positive HR-ESI-MS spectrum of compound **10**.

Figure S34. ^{1}H NMR spectrum of compound **11** in DMSO-d_6.
Figure S35. ^{13}C NMR spectrum of compound **11** in DMSO-d_6.
Figure S36. ^{1}H NMR spectrum of compound **12** in DMSO-d_6.
Figure S37. ^{13}C NMR spectrum of compound **12** in DMSO-d_6.
Figure S38. ^{1}H NMR spectrum of compound **13** in MeOD-d_4.
Figure S39. ^{13}C NMR spectrum of compound **13** in MeOD-d_4.
Figure S40. ^{1}H NMR spectrum of compound **14** in $CDCl_3$.
Figure S41. ^{13}C NMR spectrum of compound **14** in $CDCl_3$.
Figure S42. ^{1}H NMR spectrum of compound **15** in DMSO-d_6.
Figure S43. ^{13}C NMR spectrum of compound **15** in DMSO-d_6.
Figure S44. ^{1}H NMR spectrum of compound **16** in DMSO-d_6.
Figure S45. ^{13}C NMR spectrum of compound **16** in DMSO-d_6.
Figure S46. Experimental ECD spectra of compounds **1**–**5**.
Figure S47. HPLC chromatogram of compound **1** analyzed using an OptiChiral A1 column.

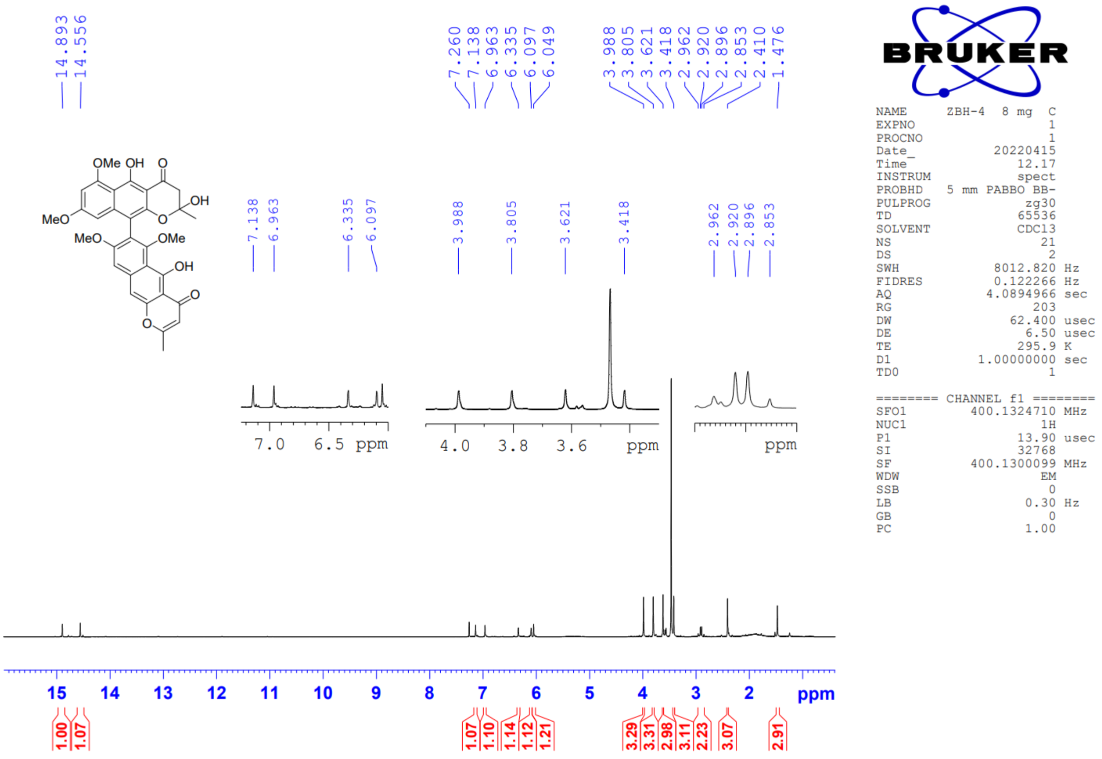

Figure S1. ^{1}H NMR spectrum of compound **1** in $CDCl_3$.

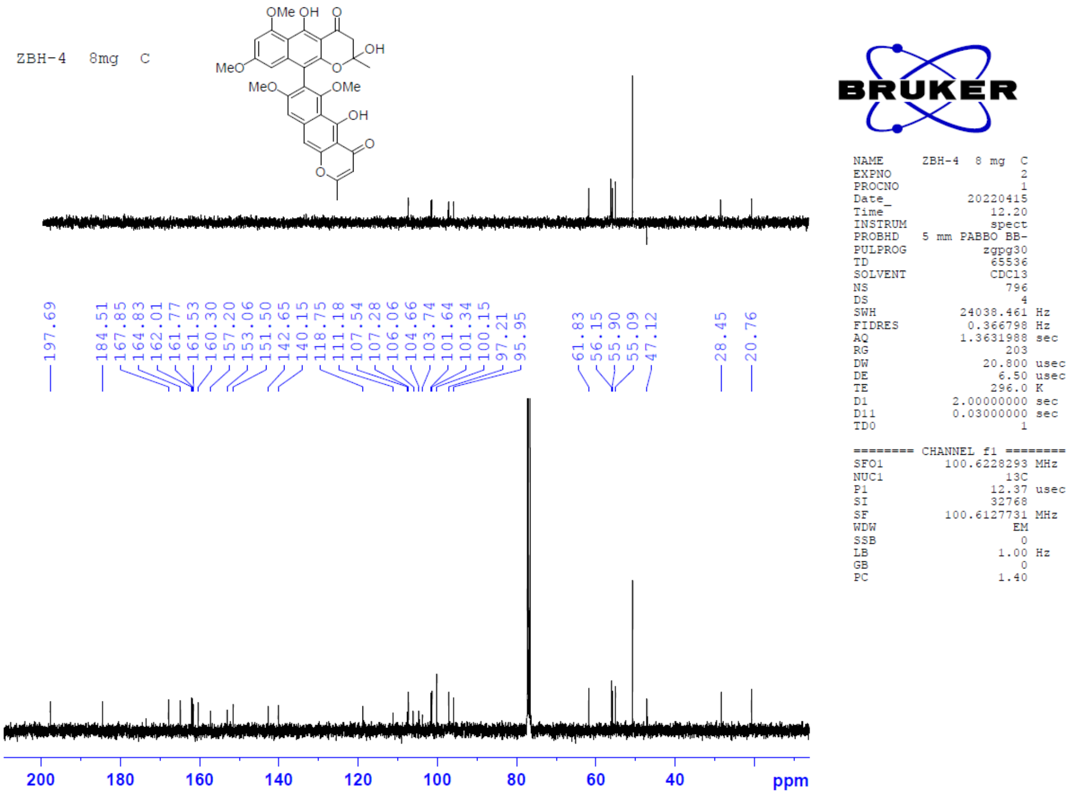

Figure S2. ^{13}C NMR spectrum of compound **1** in $CDCl_3$.

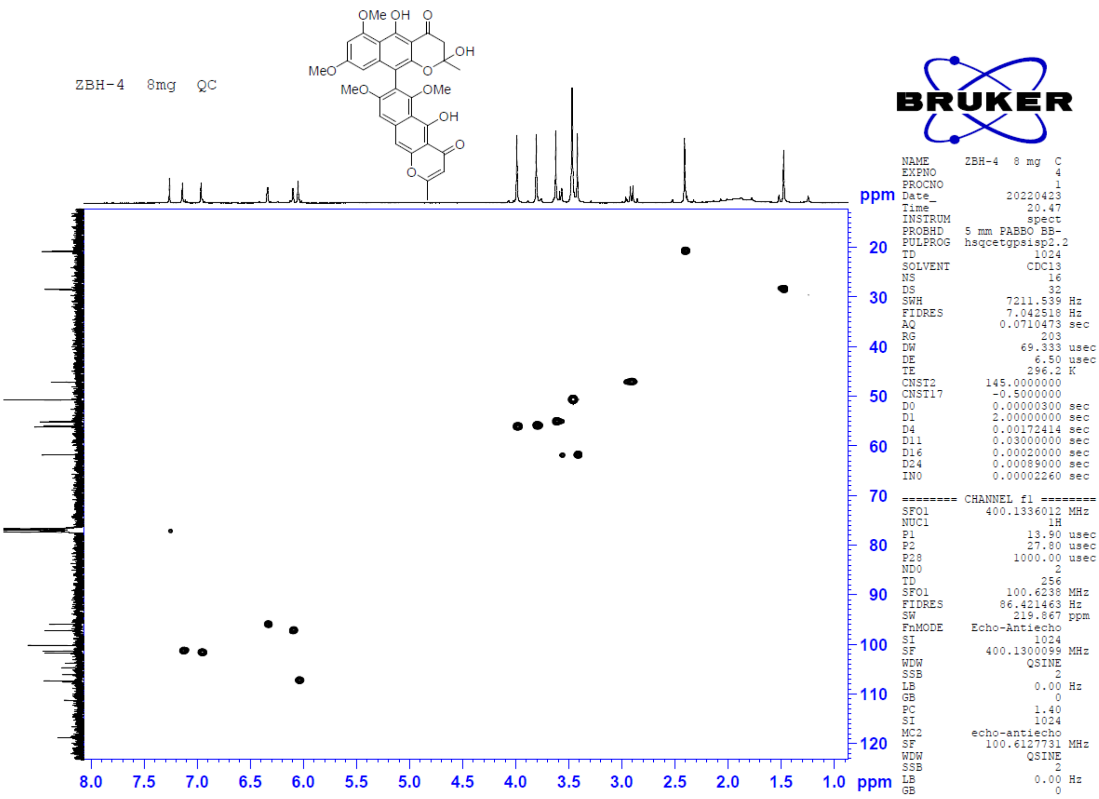

Figure S3. HSQC spectrum of compound **1** in $CDCl_3$.

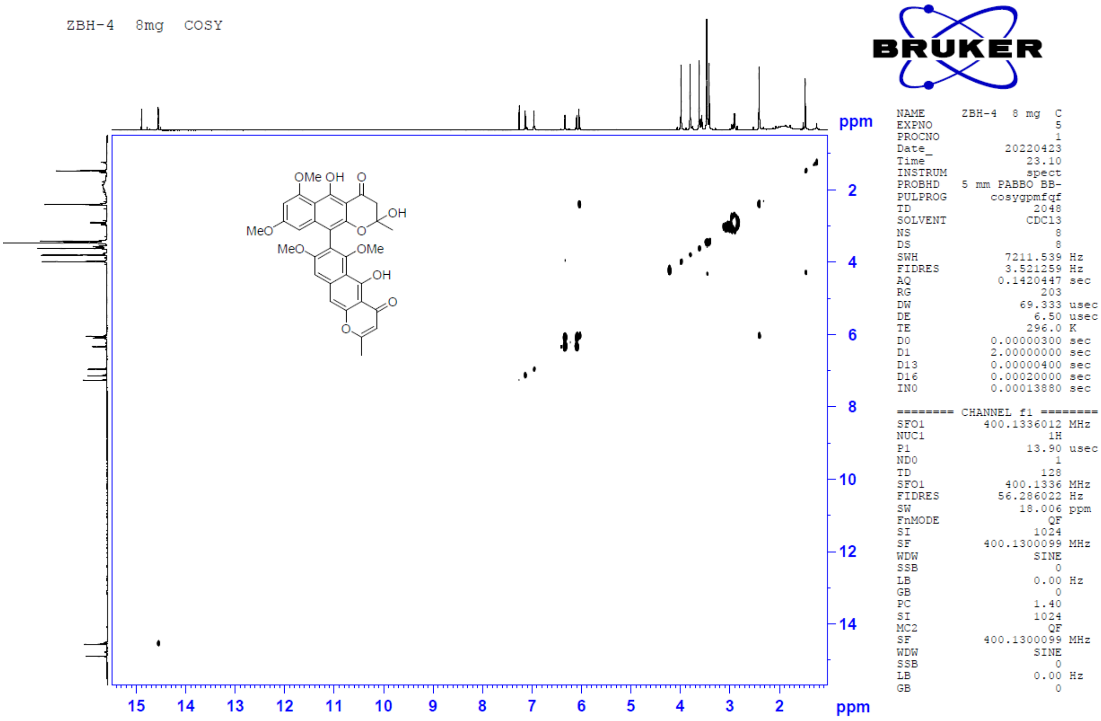

Figure S4. ^{1}H-^{1}H COSY spectrum of compound **1** in $CDCl_3$.

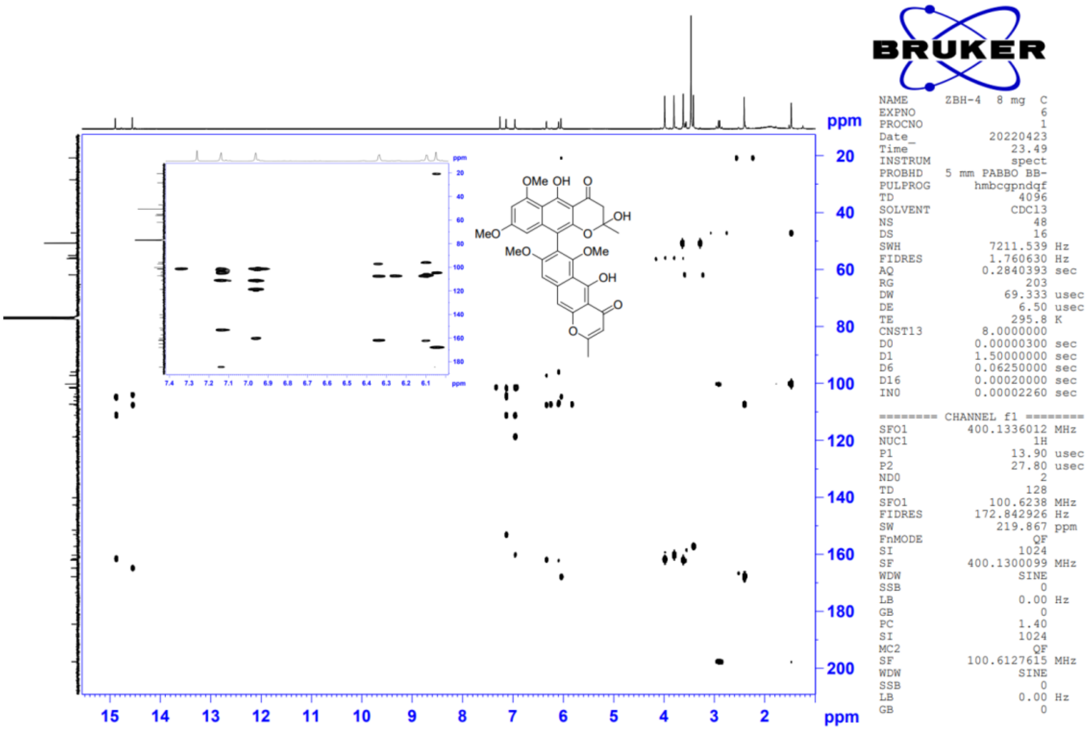

Figure S5. HMBC spectrum of compound **1** in $CDCl_3$.

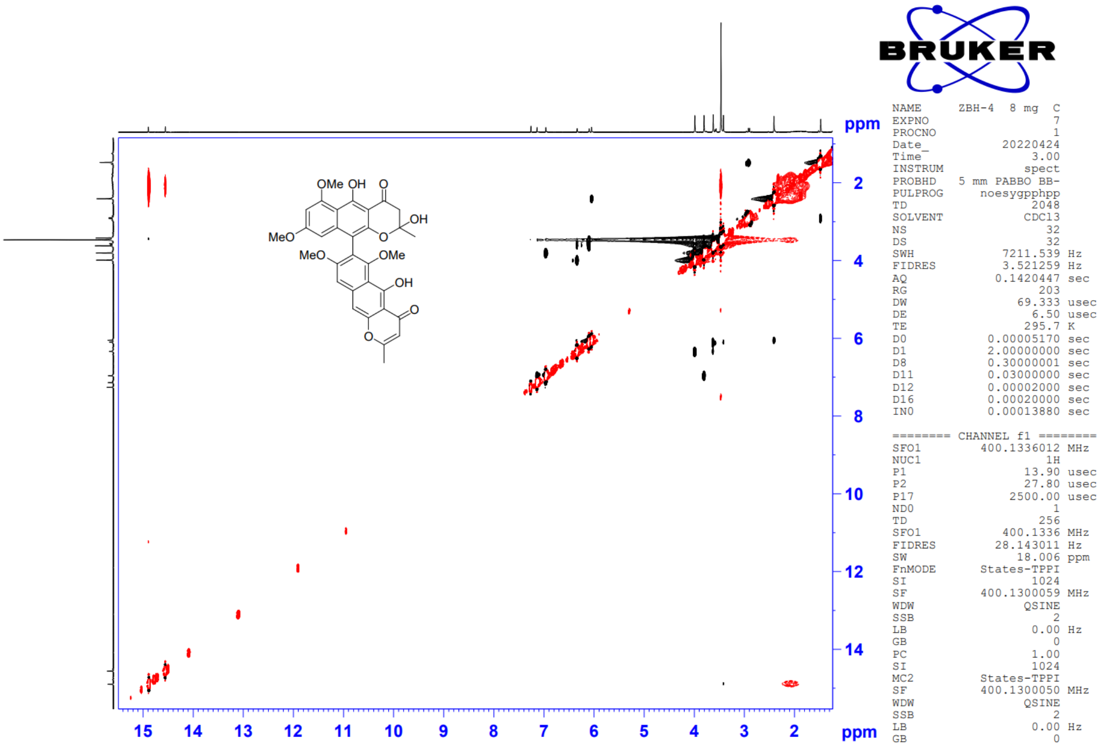

Figure S6. NOESY spectrum of compound **1** in $CDCl_3$.

Elemental Composition Report Page 1

Tolerance = 50.0 PPM / DBE: min = -1.5, max = 50.0
Element prediction: Off
Number of isotope peaks used for i-FIT = 3

Monoisotopic Mass, Even Electron Ions
30 formula(e) evaluated with 1 results within limits (up to 50 best isotopic matches for each mass)
Elements Used:
C: 5-35 H: 0-60 O: 0-10 23Na: 0-1
ZBH-4 70 (0.290) Cm (69:72)
1: TOF MS ES+

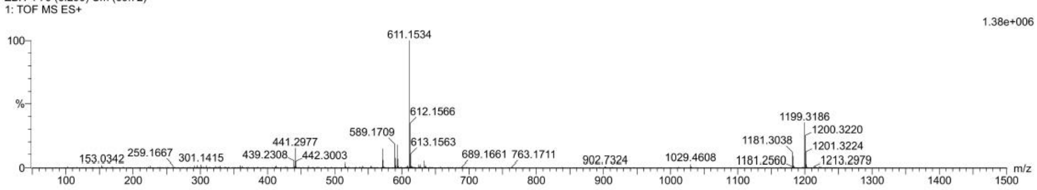

Minimum:	80.00				-1.5				
Maximum:	100.00		50.0	50.0	50.0				
Mass	RA	Calc. Mass	mDa	PPM	DBE	i-FIT	Norm	Conf(%)	Formula
611.1534	100.00	611.1318	21.6	35.3	23.5	1220.4	n/a	n/a	C35 H24 O9 23Na

Figure S7. The positive HR-ESI-MS spectrum of compound **1**.

Anton Paar GmbH
Anton Paar Strasse 10
8054 Graz
Austria

Anton Paar Polarimeter - Measurement(s)

MCP 100
Software Version: 1.50.4098.87
Serial Number: 99032784

Unique Id 2206

- Sample Name: ZBH-4
- Date: 09/27/2023 - 09:15 AM
- Username: Administrator
- Sample State: Ok

- Measurement Mode: Specific Rotation
- Measurement Result: -12.000 °
- Concentration: 0.100 g/100ml
- Optical Rotation: -0.012 °
- Set Temperature: 25.0 °C
- Temperature: 25.0 °C

Figure S8. Optical rotation spectrum of compound **1**.

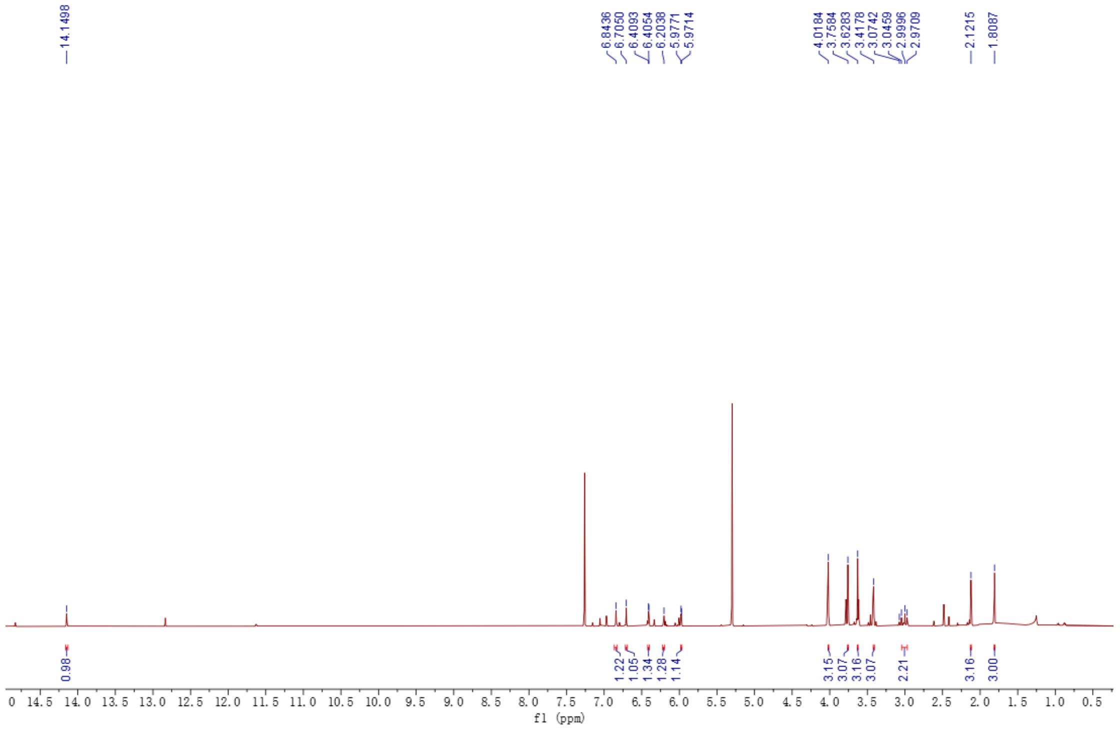

Figure S9. ^{1}H NMR spectrum of compound **2** in $CDCl_3$.

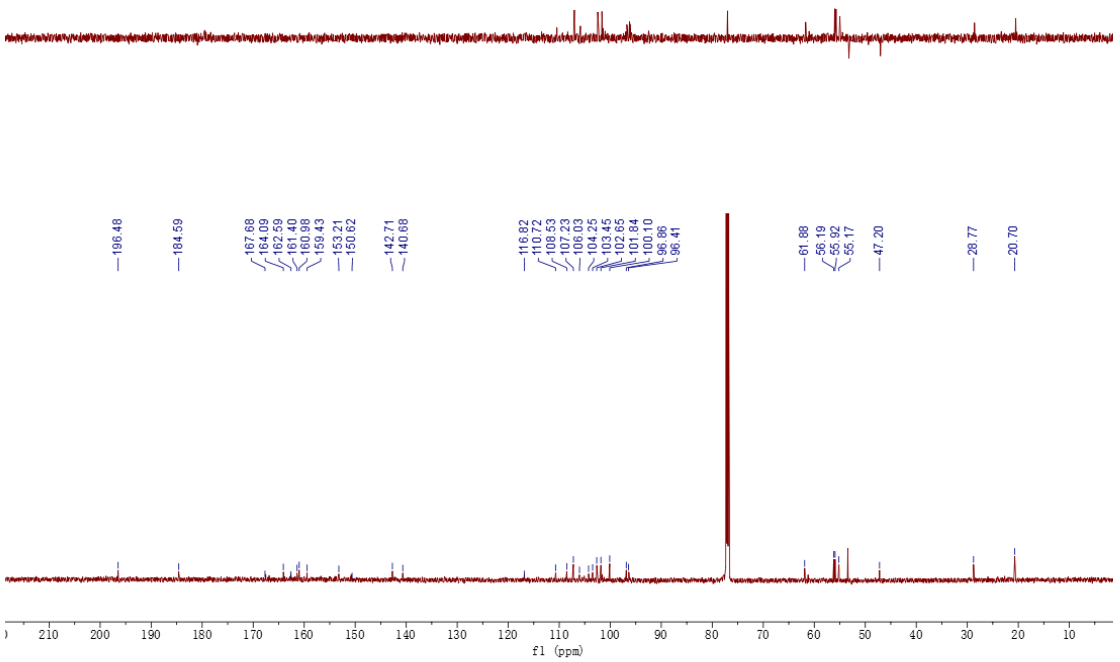

Figure S10. ^{13}C NMR spectrum of compound **2** in $CDCl_3$.

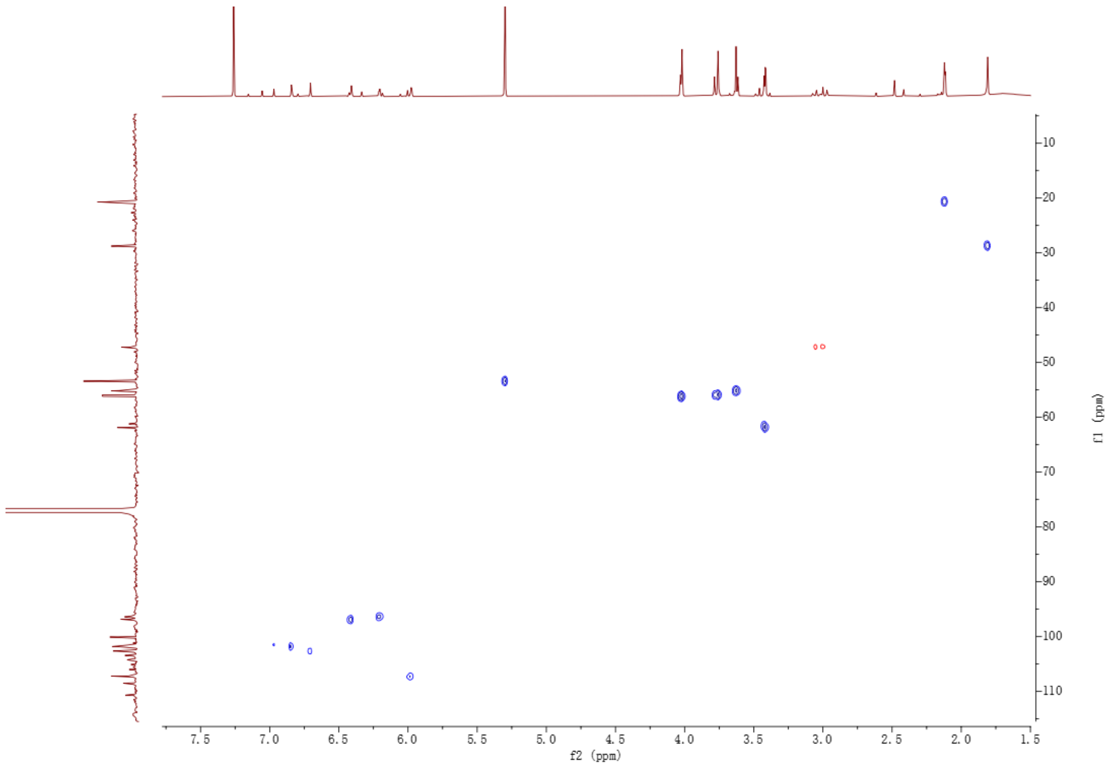

Figure S11. HSQC spectrum of compound **2** in $CDCl_3$.

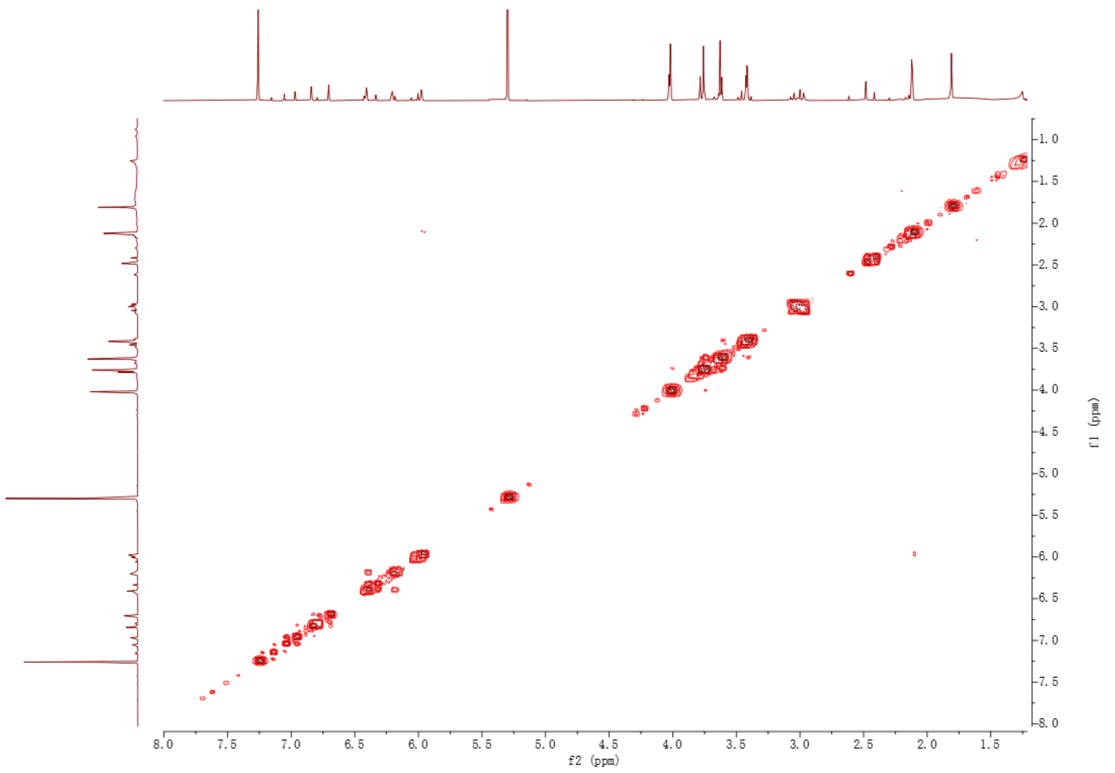

Figure S12. ^{1}H-^{1}H COSY spectrum of compound **2** in $CDCl_3$.

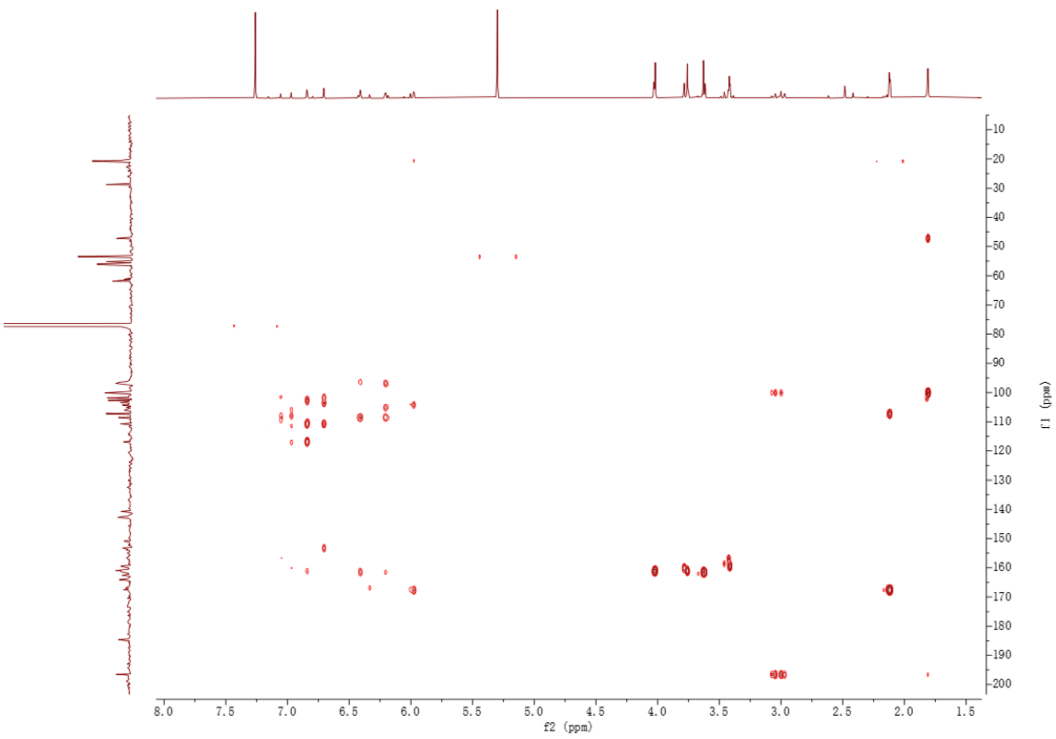

Figure S13. HMBC spectrum of compound **2** in $CDCl_3$.

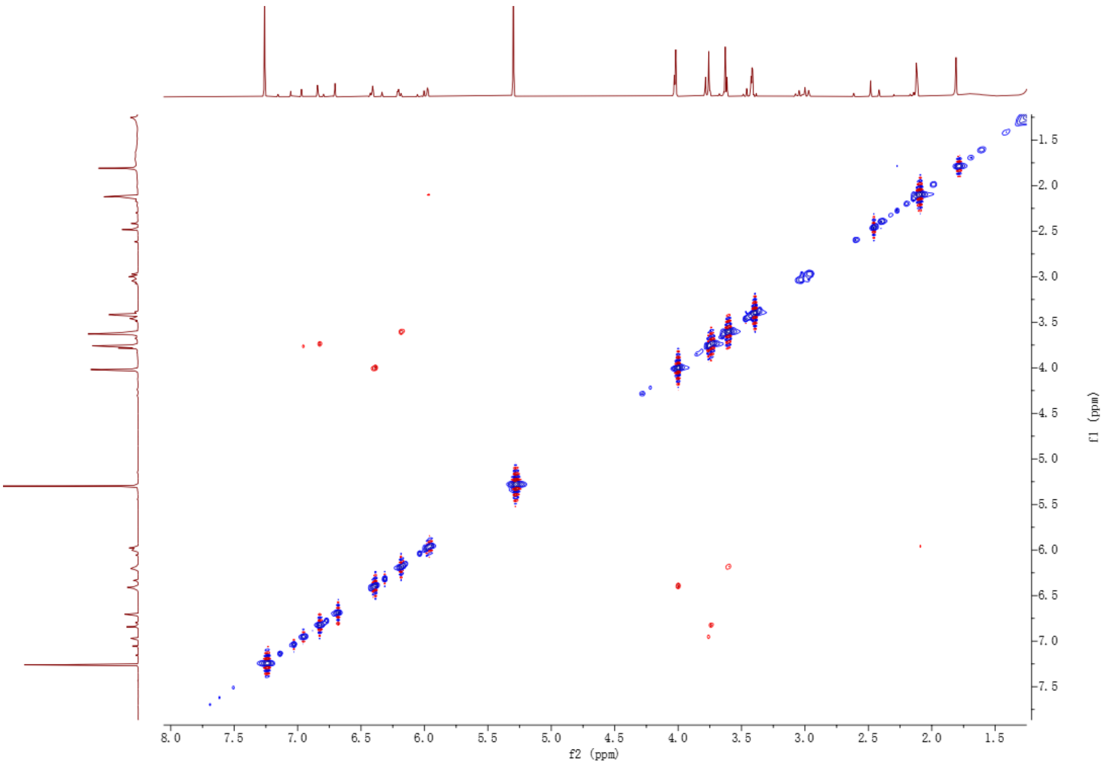

Figure S14. NOESY spectrum of compound **2** in $CDCl_3$.

Elemental Composition Report **Page 1**

Single Mass Analysis
Tolerance = 50.0 mDa / DBE: min = -1.5, max = 50.0
Element prediction: Off
Number of isotope peaks used for i-FIT = 3

Monoisotopic Mass, Even Electron Ions
82 formula(e) evaluated with 14 results within limits (up to 50 closest results for each mass)
Elements Used:
C: 5-35 H: 0-60 O: 0-15 Na: 0-1
ZBH-3A 70 (0.290) Cm (63:95)
1: TOF MS ES+

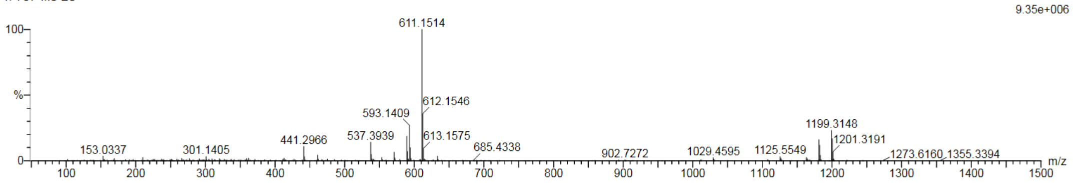

Minimum:				-1.5				
Maximum:		50.0	10.0	50.0				

Mass	Calc. Mass	mDa	PPM	DBE	i-FIT	Norm	Conf(%)	Formula
611.1514	611.1529	-1.5	-2.5	18.5	1990.5	1.443	23.63	C32 H28 O11 Na
	611.1553	-3.9	-6.4	21.5	1990.6	1.490	22.54	C34 H27 O11
	611.1401	11.3	18.5	17.5	1992.7	3.651	2.60	C30 H27 O14
	611.1377	13.7	22.4	14.5	1995.4	6.323	0.18	C28 H28 O14 Na
	611.1318	19.6	32.1	23.5	1993.0	3.940	1.95	C35 H24 O9 Na
	611.1741	-22.7	-37.1	13.5	1995.2	6.102	0.22	C29 H32 O13 Na
	611.1765	-25.1	-41.1	16.5	1992.1	2.991	5.03	C31 H31 O13
	611.1190	32.4	53.0	22.5	1990.0	0.918	39.93	C33 H23 O12
	611.1165	34.9	57.1	19.5	1993.8	4.753	0.86	C31 H24 O12 Na
	611.1893	-37.9	-62.0	17.5	1992.7	3.611	2.70	C33 H32 O10 Na
	611.1917	-40.3	-65.9	20.5	1994.9	5.774	0.31	C35 H31 O10
	611.1952	-43.8	-71.7	8.5	1999.3	10.262	0.00	C26 H36 O15 Na
	611.1976	-46.2	-75.6	11.5	1997.9	8.770	0.02	C28 H35 O15
	611.1037	47.7	78.0	18.5	1996.9	7.834	0.04	C29 H23 O15

Figure S15. The positive HR-ESI-MS spectrum of compound **2**.

Anton Paar GmbH
Anton Paar Strasse 10
8054 Graz
Austria

Anton Paar

Anton Paar Polarimeter - Measurement(s)

MCP 100
Software Version: 1.50.4366.160
Serial Number: 99154608

Unique Id 1406

- Sample Name: ZBH-3A
- Date: 03/19/2026 - 07:58 AM
- Username: Administrator
- Sample State: Ok

- Measurement Mode: Specific Rotation
- Measurement Result: -41.000 °
- Concentration: 0.300 g/100ml
- Optical Rotation: -0.123 °
- Set Temperature: 25.0 °C
- Temperature: 25.0 °C

Figure S16. Optical rotation spectrum of compound **2**.

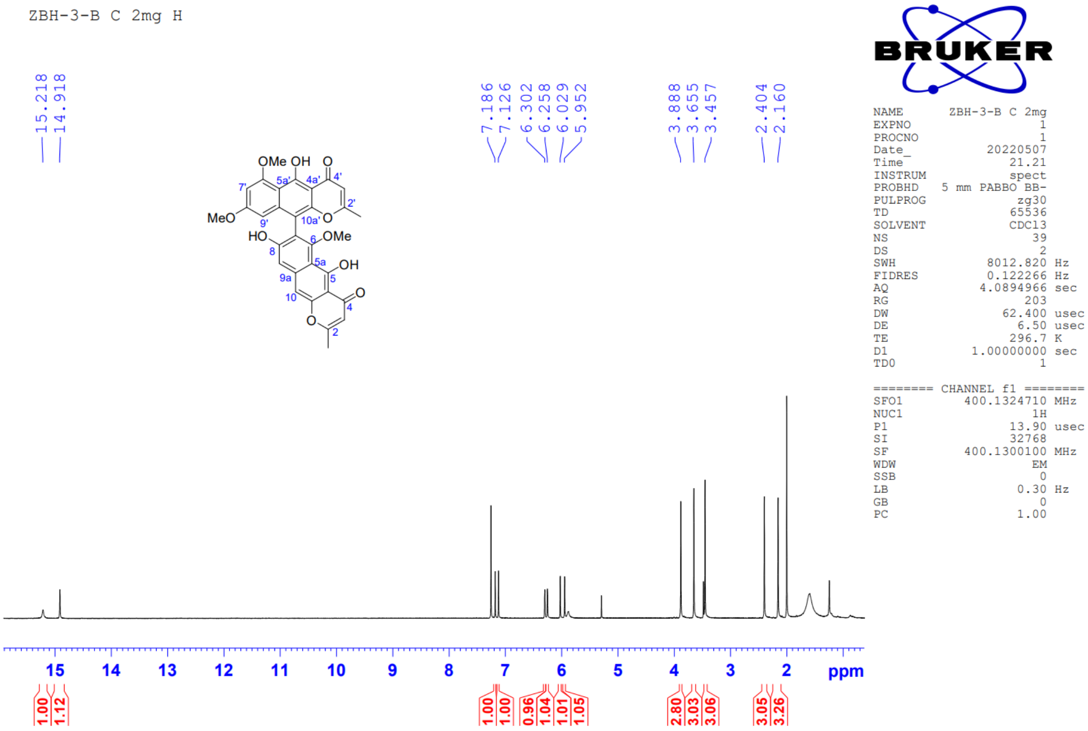

Figure S17. ^{1}H NMR spectrum of compound **3** in $CDCl_3$.

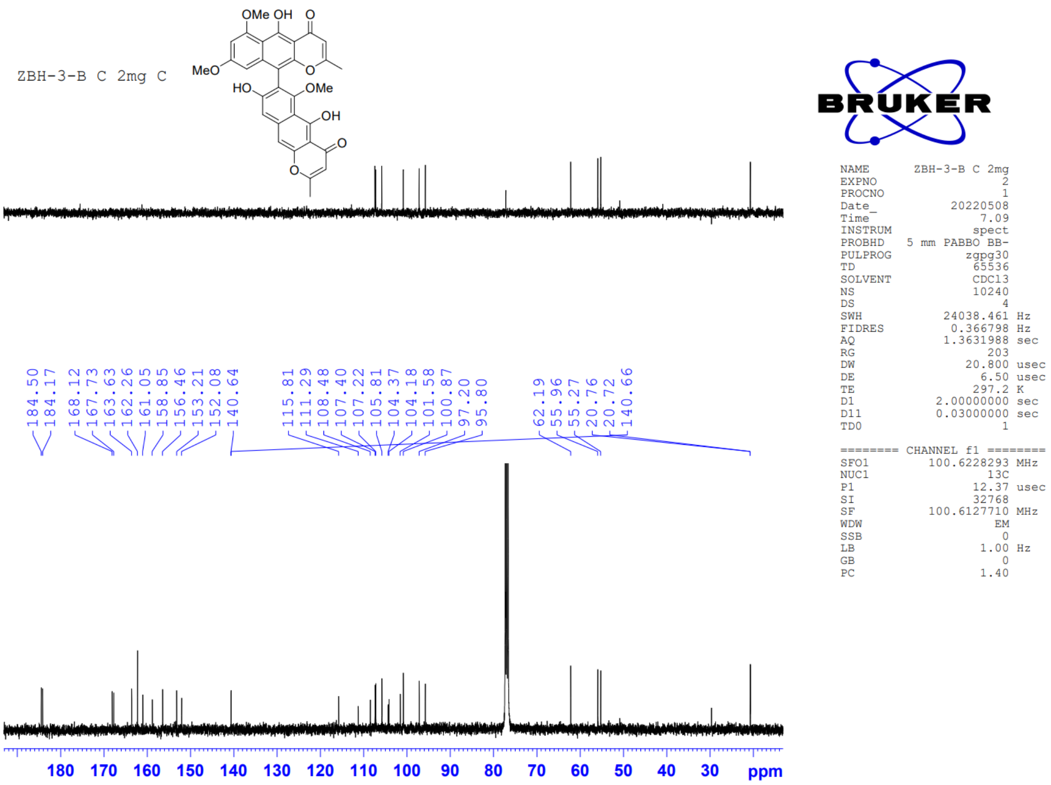

Figure S18. ^{13}C NMR spectrum of compound **3** in $CDCl_3$.

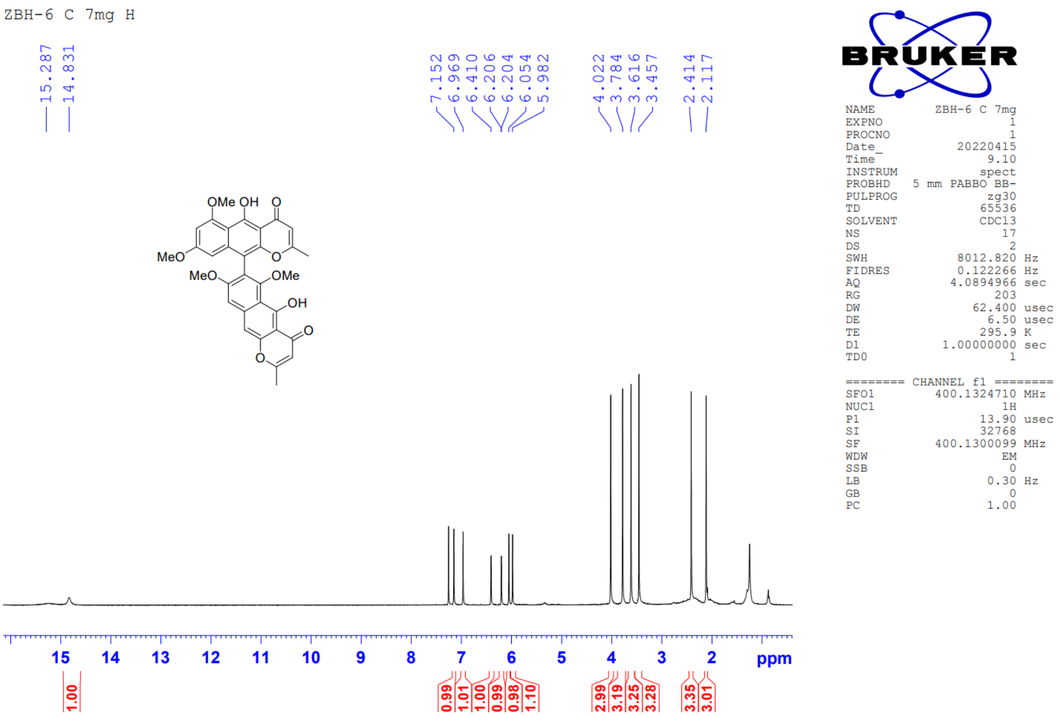

Figure S19. ^{1}H NMR spectrum of compound **4** in $CDCl_3$.

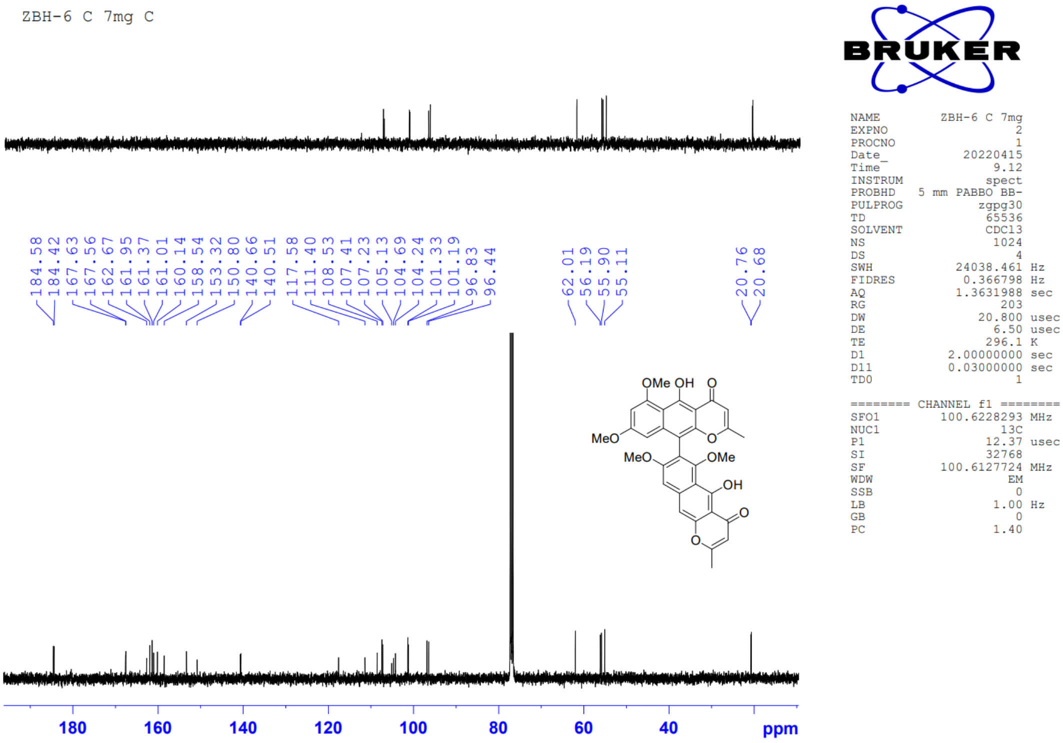

Figure S20. ^{13}C NMR spectrum of compound **4** in $CDCl_3$.

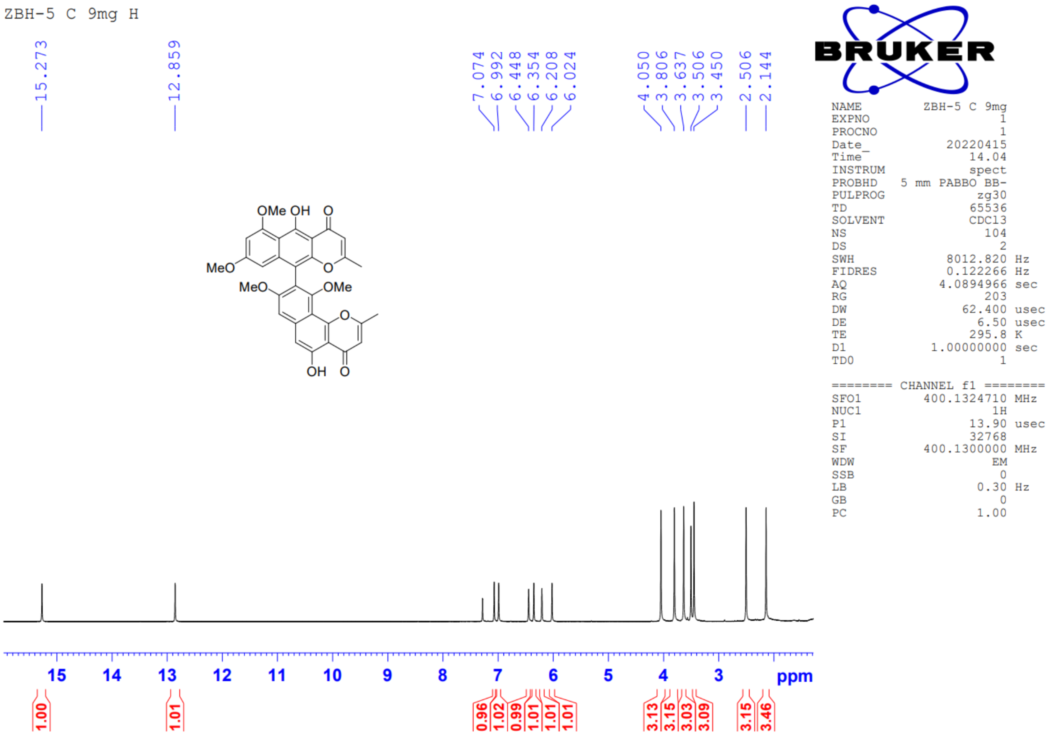

Figure S21. ^{1}H NMR spectrum of compound **5** in $CDCl_3$.

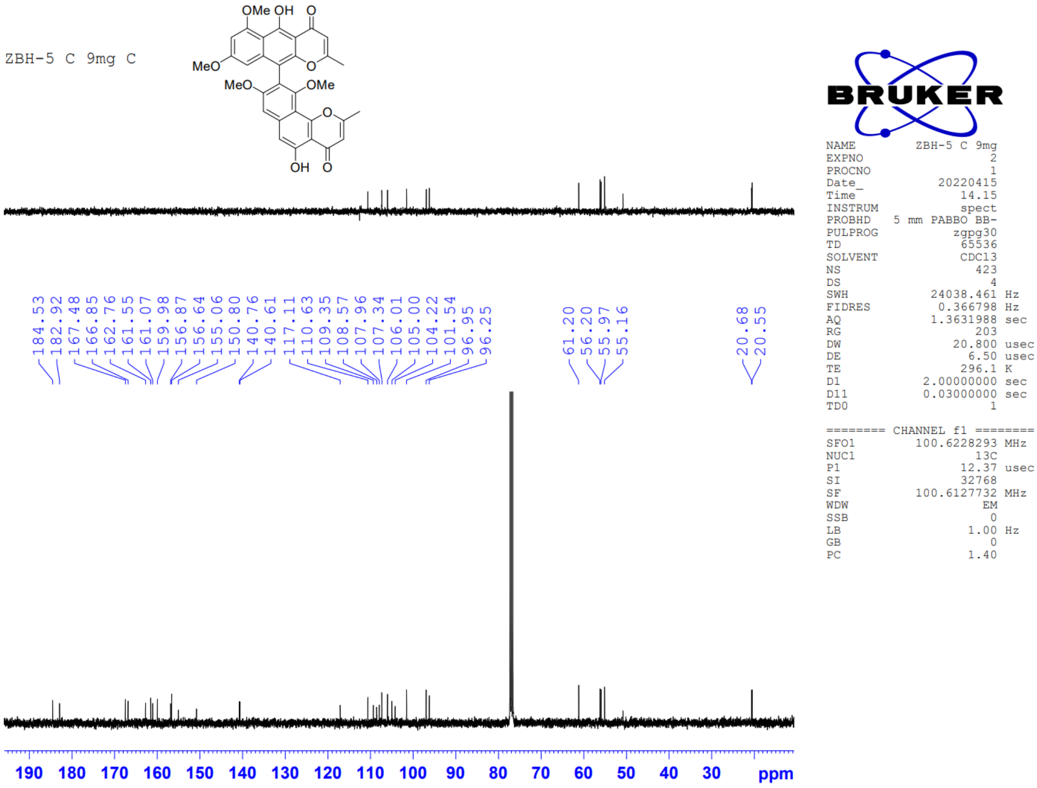

Figure S22. ^{13}C NMR spectrum of compound **5** in $CDCl_3$.

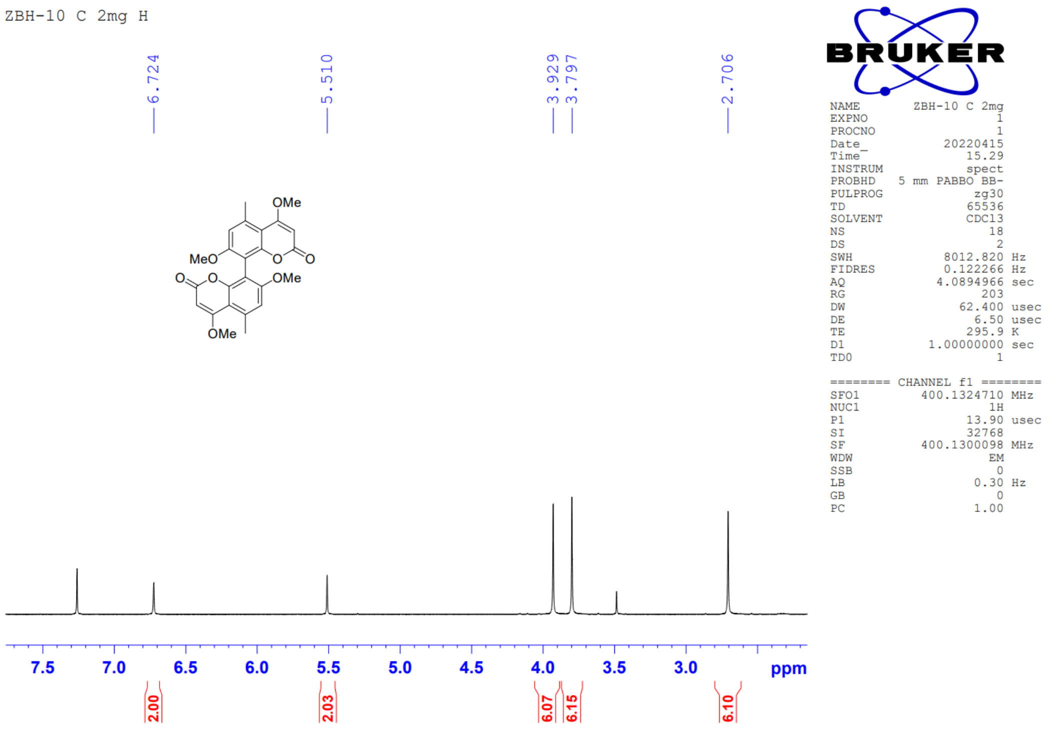

Figure S23. ^{1}H NMR spectrum of compound **6** in $CDCl_3$.

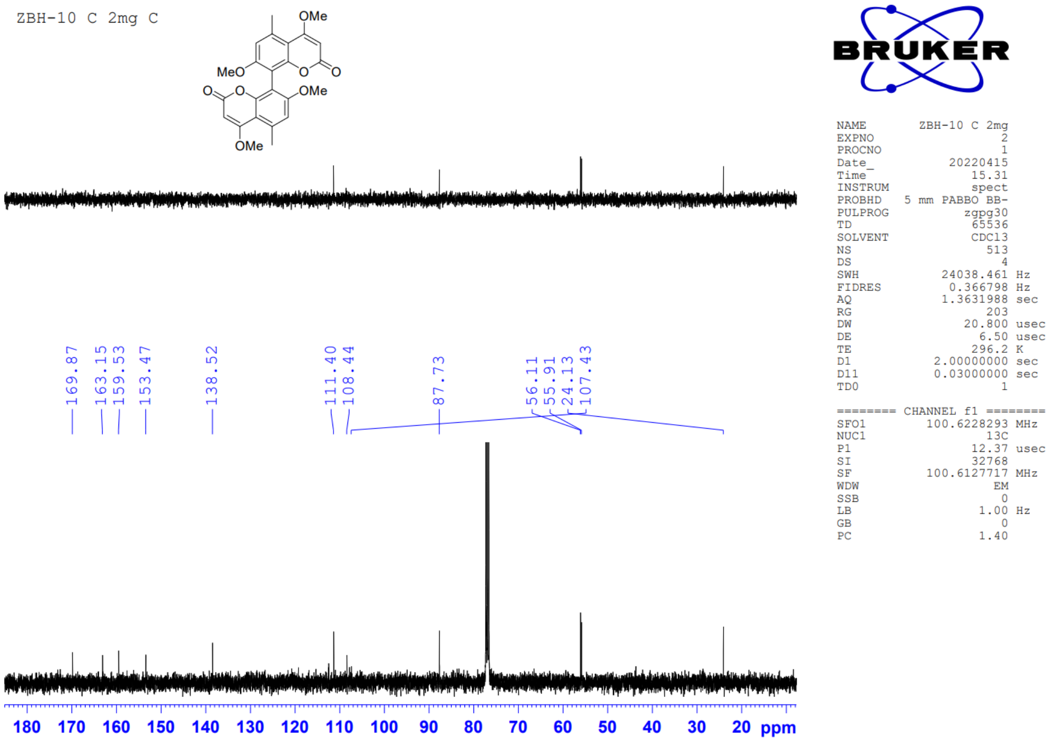

Figure S24. ^{13}C NMR spectrum of compound **6** in $CDCl_3$.

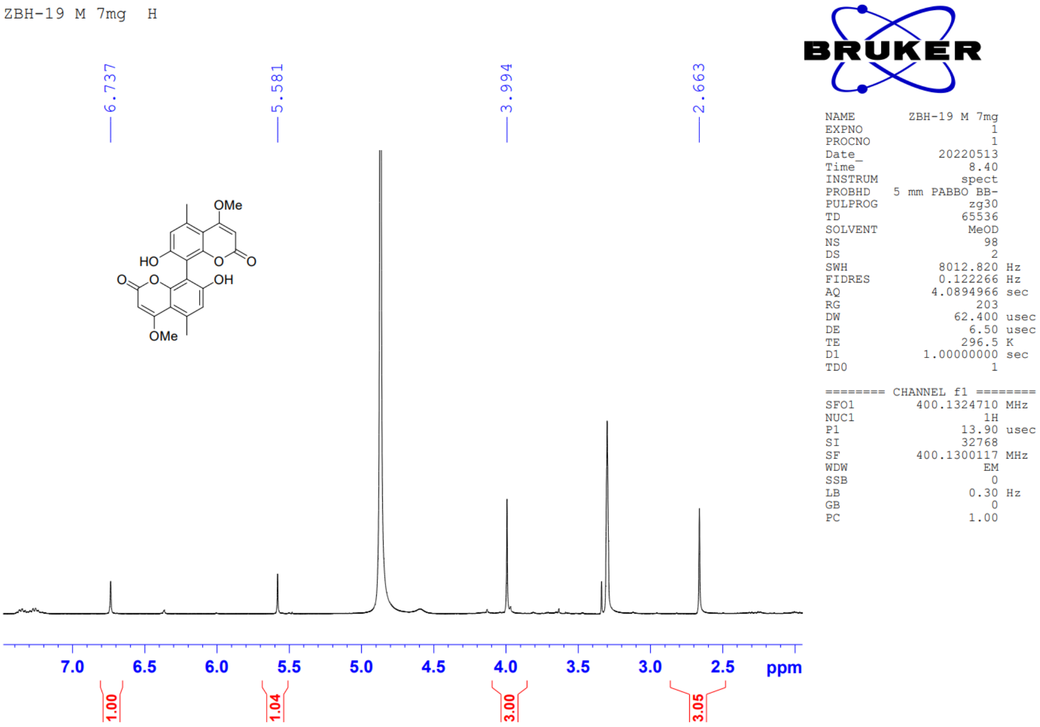

Figure S25. ^{1}H NMR spectrum of compound **7** in MeOD-*d*$_4$.

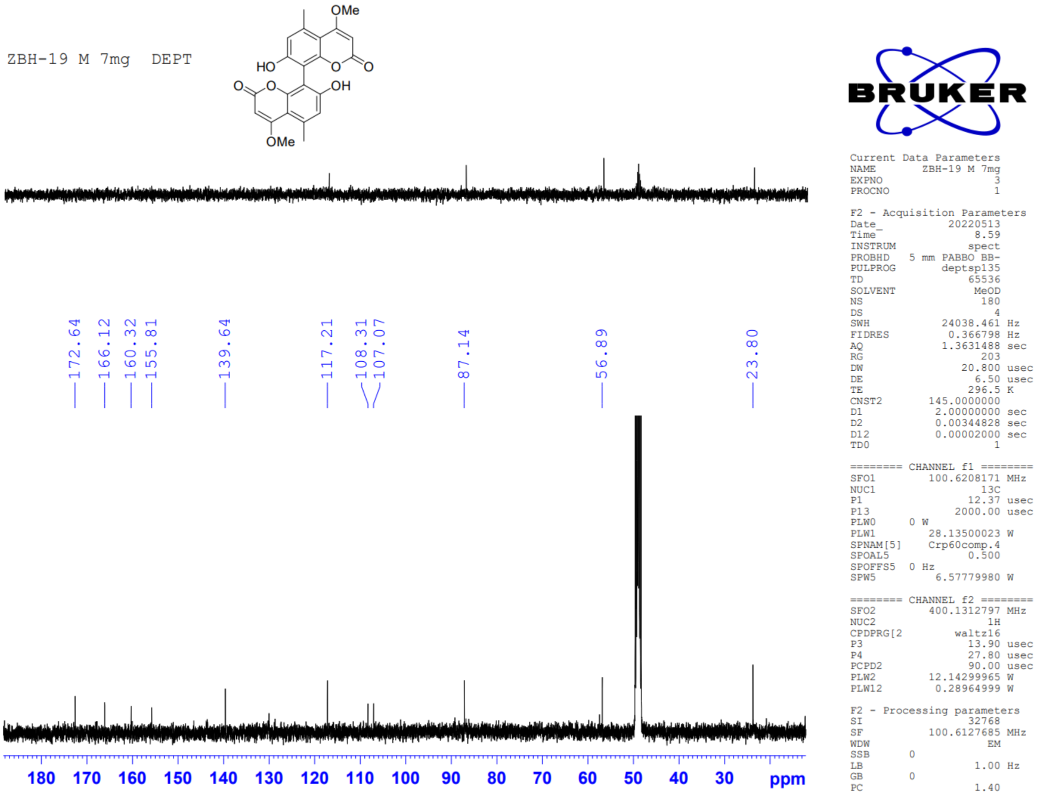

Figure S26. ^{13}C NMR spectrum of compound **7** in MeOD-*d*$_4$.

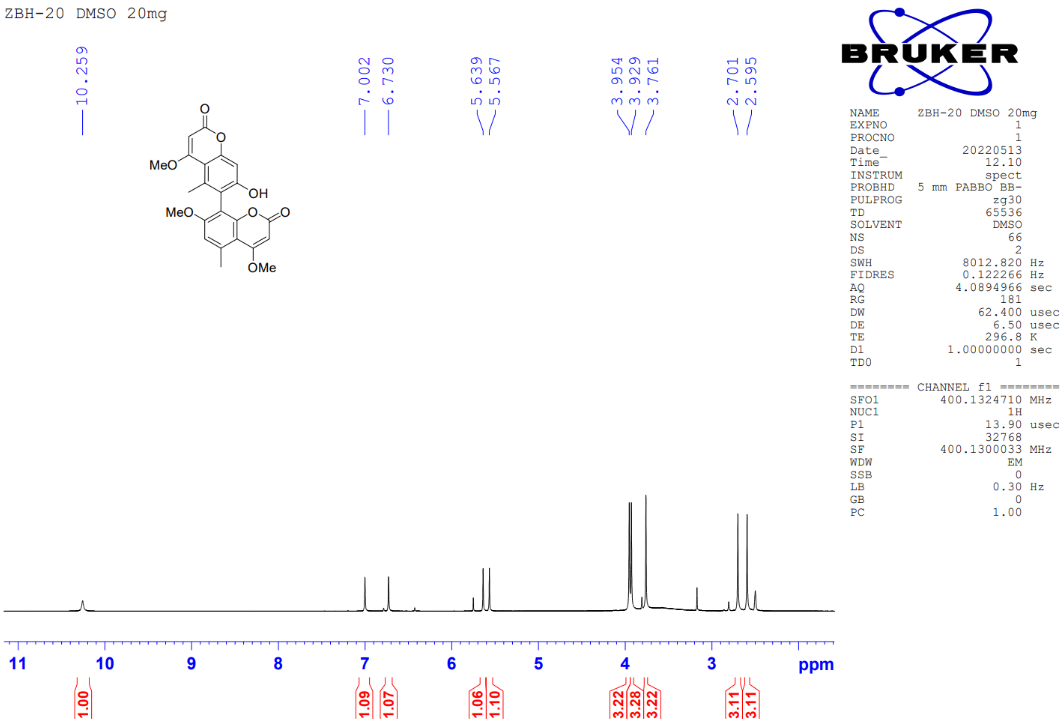

Figure S27. ^{1}H NMR spectrum of compound **8** in DMSO-d_6.

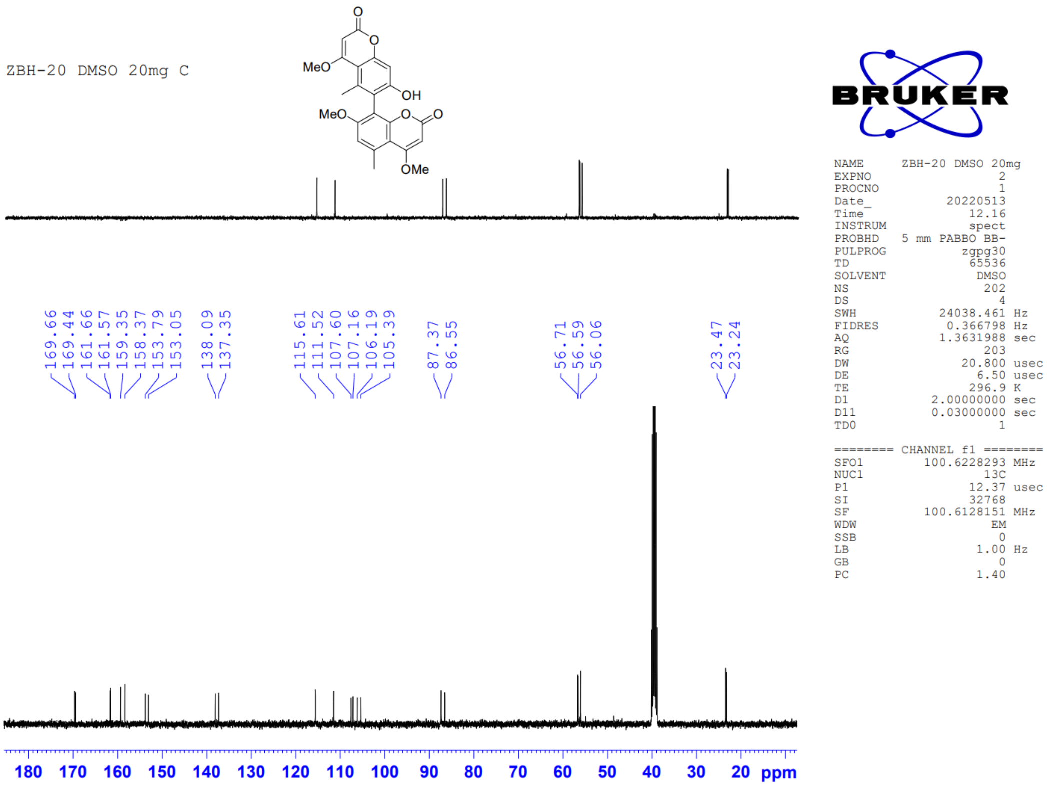

Figure S28. ^{13}C NMR spectrum of compound **8** in DMSO-d_6.

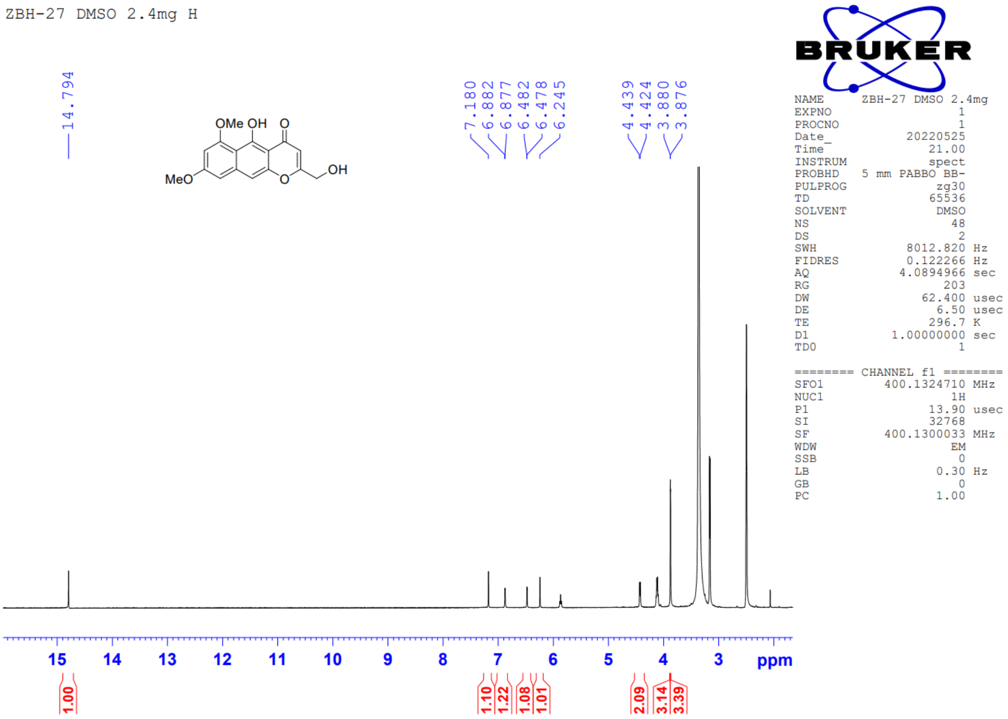

Figure S29. ^{1}H NMR spectrum of compound **9** in DMSO-d_6.

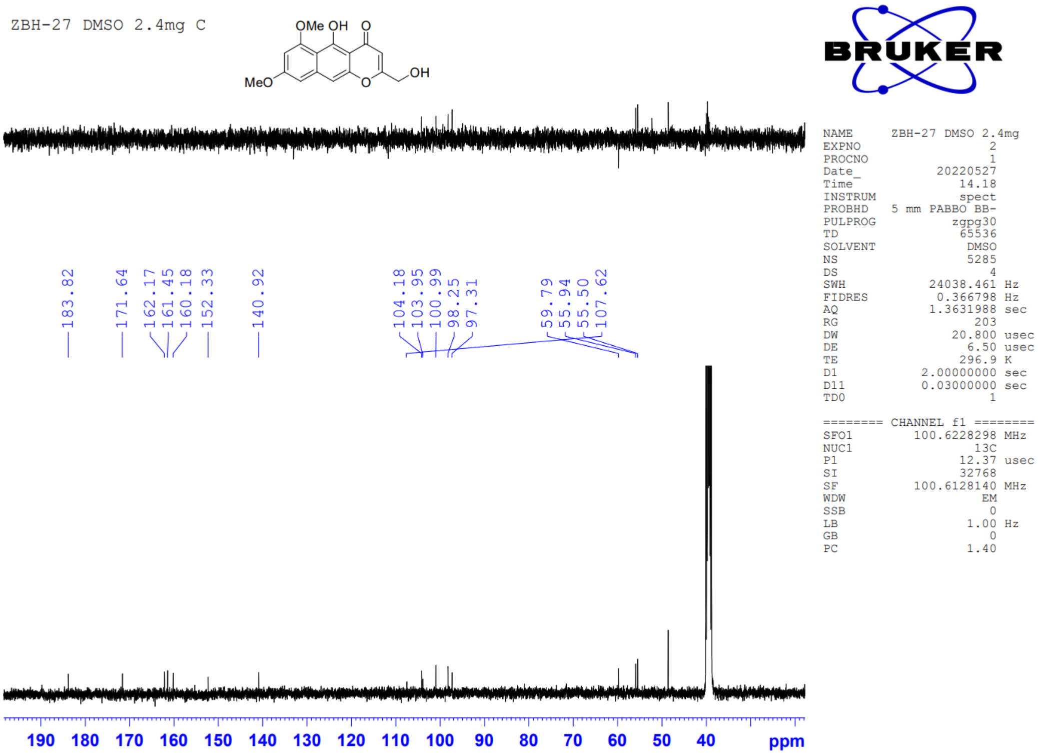

Figure S30. ^{13}C NMR spectrum of compound **9** in DMSO-d_6.

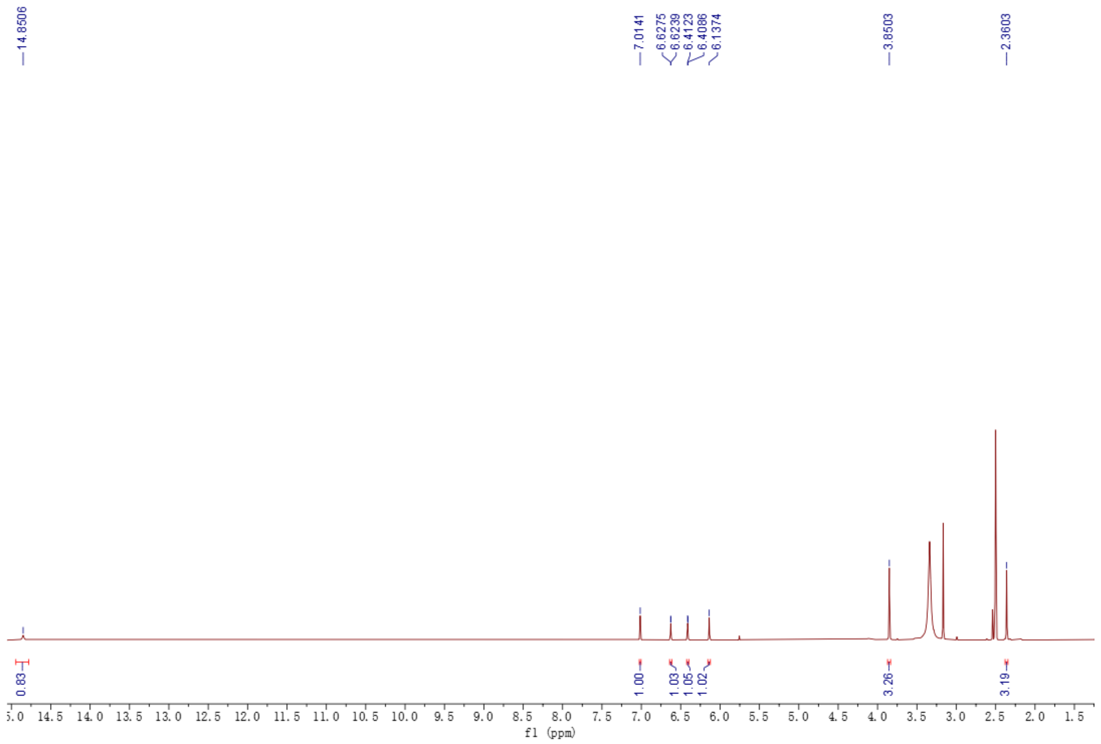

Figure S31. ^{1}H NMR spectrum of compound **10** in DMSO-d_6.

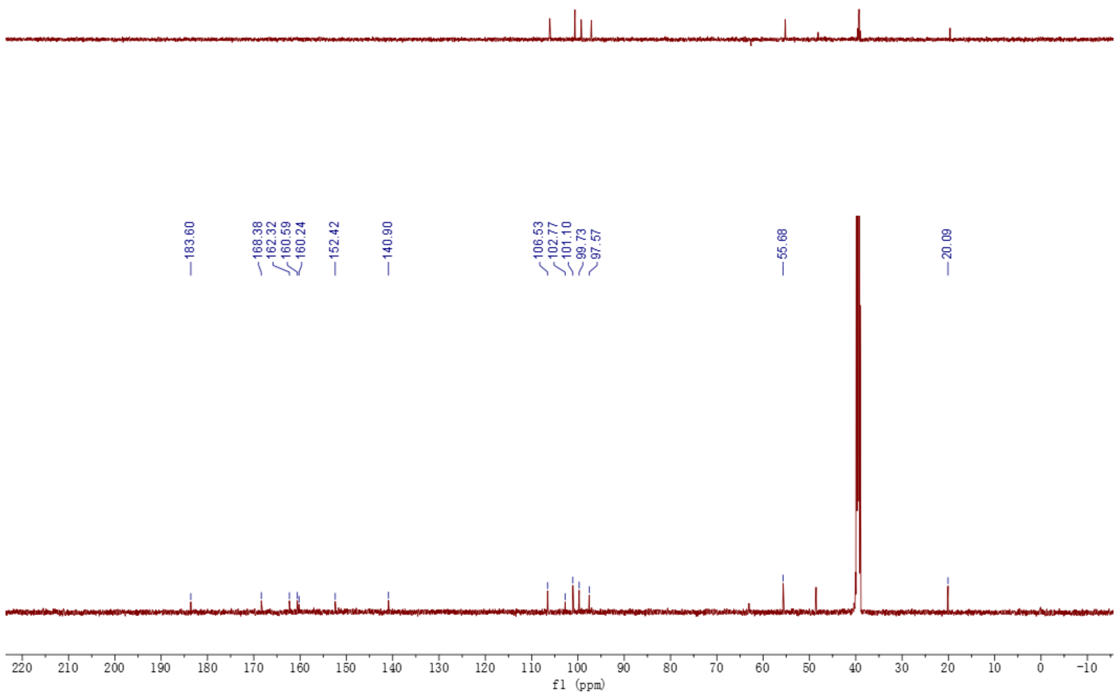

Figure S32. ^{13}C NMR spectrum of compound **10** in DMSO-d_6.

Elemental Composition Report **Page 1**

Tolerance = 50.0 PPM / DBE: min = -1.5, max = 50.0
Element prediction: Off
Number of isotope peaks used for i-FIT = 3

Monoisotopic Mass, Even Electron Ions
75 formula(e) evaluated with 8 results within limits (up to 50 best isotopic matches for each mass)
Elements Used:
C: 5-35 H: 0-60 O: 0-10 23Na: 0-1
ZBH-1 70 (0.290) Cm (70:72)
1: TOF MS ES+

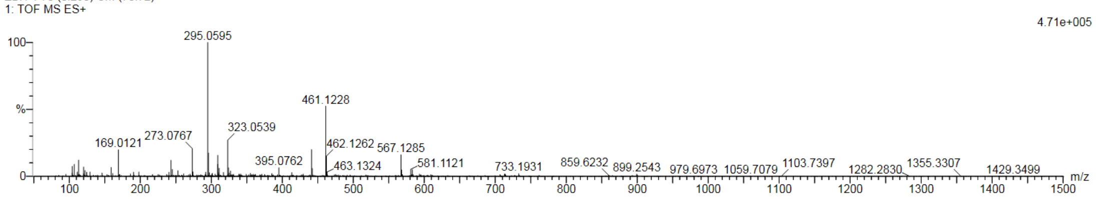

Minimum:	80.00				-1.5				
Maximum:	100.00		50.0	50.0	50.0				

Mass	RA	Calc. Mass	mDa	PPM	DBE	i-FIT	Norm	Conf(%)	Formula
295.0595	100.00	295.0582	1.3	4.4	9.5	992.6	0.347	70.66	C15 H12 O5 23Na
		295.0606	-1.1	-3.7	12.5	993.5	1.230	29.23	C17 H11 O5
		295.0454	14.1	47.8	8.5	999.9	7.576	0.05	C13 H11 O8
		295.0735	-14.0	-47.4	13.5	999.9	7.618	0.05	C19 H12 O2 23Na
		295.0524	7.1	24.1	18.5	1002.3	9.994	0.00	C22 H8 23Na
		295.0548	4.7	15.9	21.5	1003.0	10.712	0.00	C24 H7
		295.0665	-7.0	-23.7	3.5	1003.2	10.871	0.00	C10 H15 O10
		295.0641	-4.6	-15.6	0.5	1004.7	12.463	0.00	C8 H16 O10 23Na

Figure S33. The positive HR-ESI-MS spectrum of compound **10**.

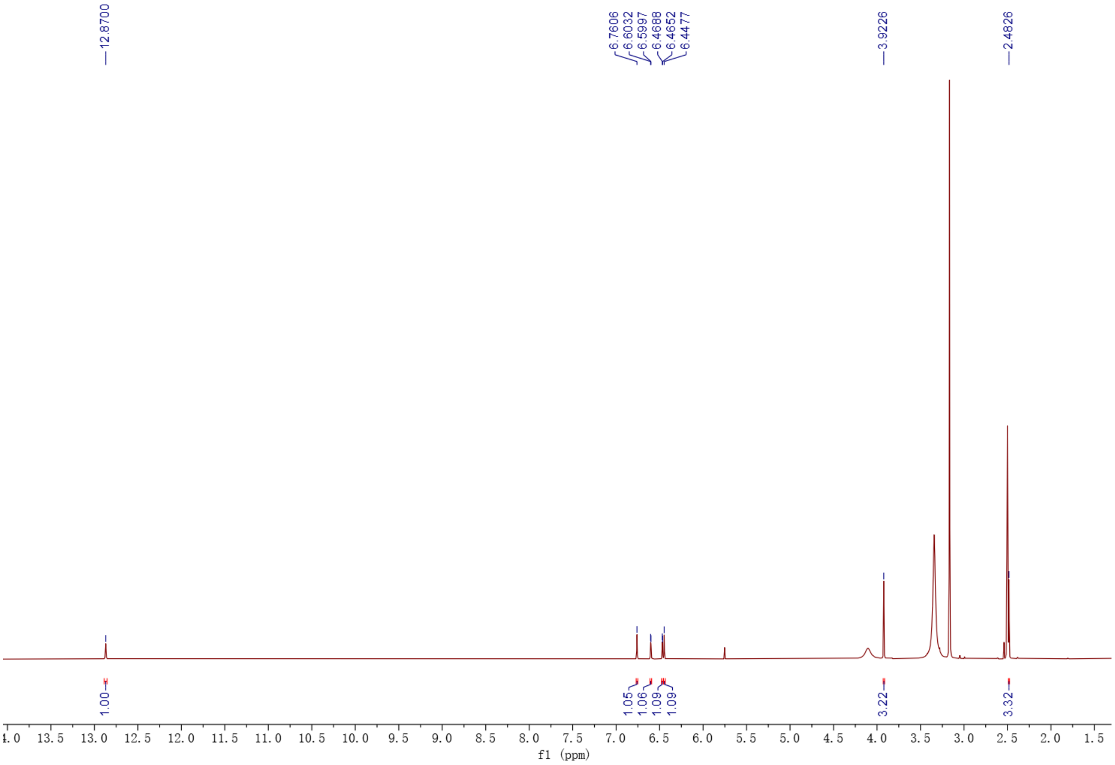

Figure S34. ^{1}H NMR spectrum of compound **11** in DMSO-d_6.

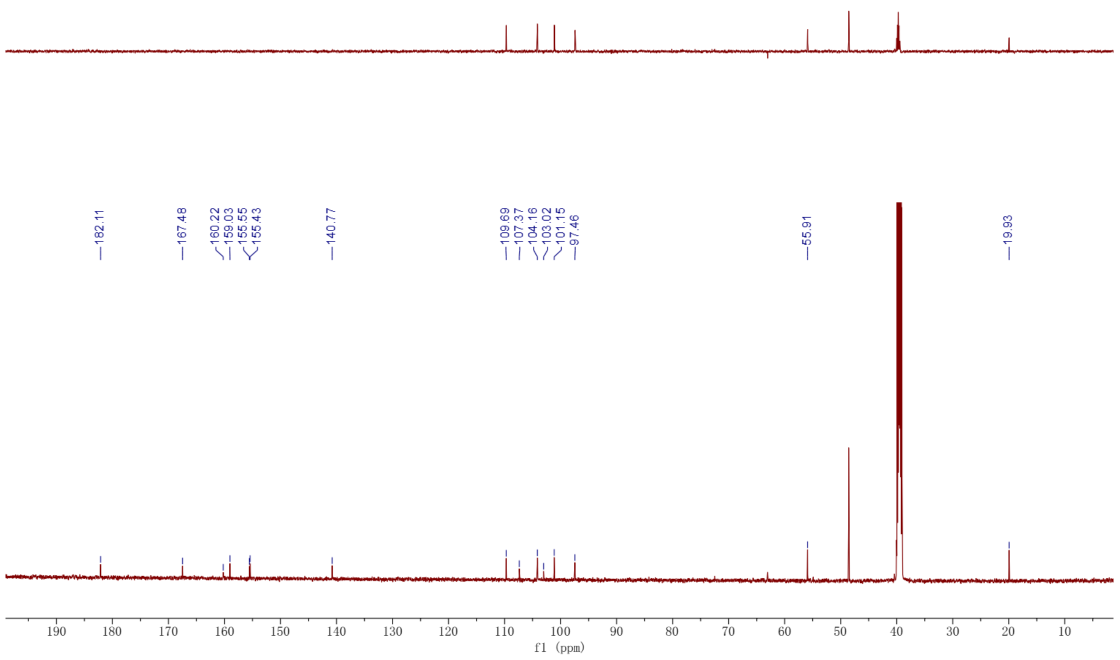

Figure S35. ^{13}C NMR spectrum of compound **11** in DMSO-d_6.

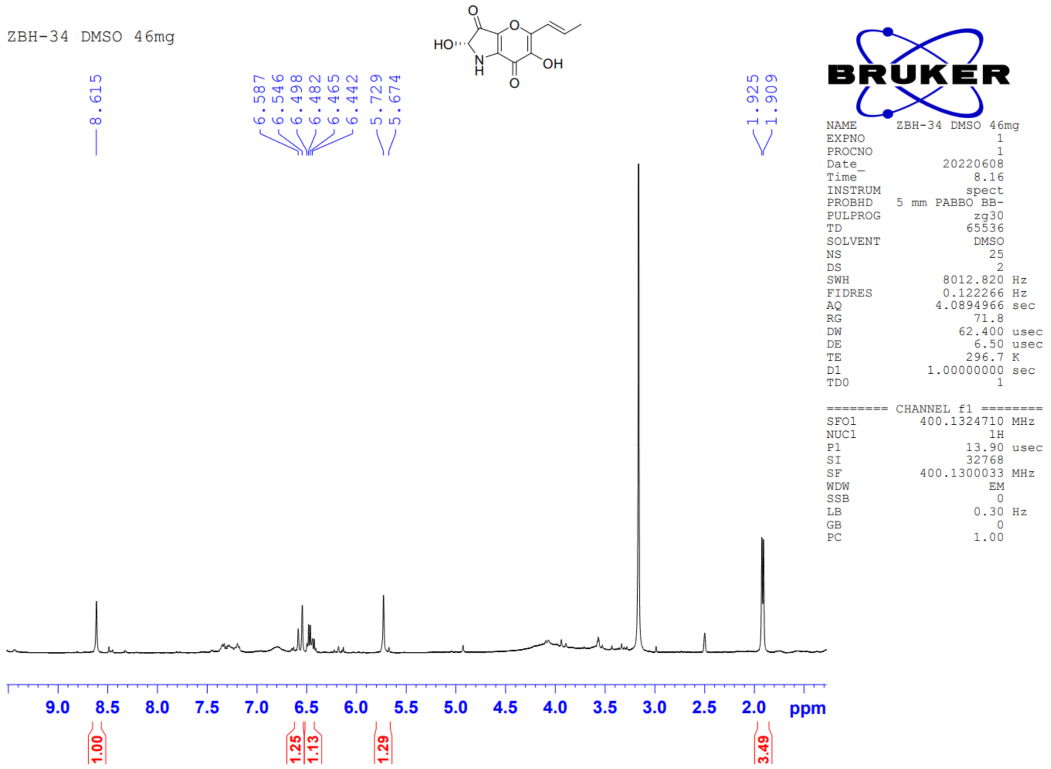

Figure S36. ^{1}H NMR spectrum of compound **12** in DMSO-d_6.

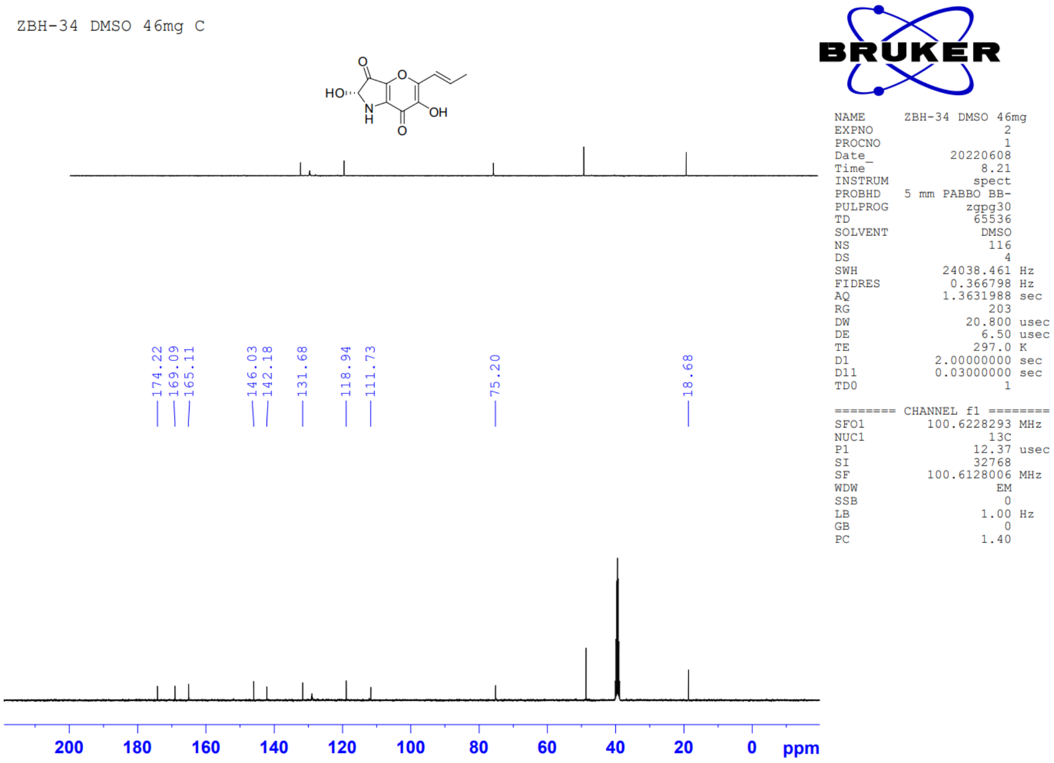

Figure S37. ^{13}C NMR spectrum of compound **12** in DMSO-d_6.

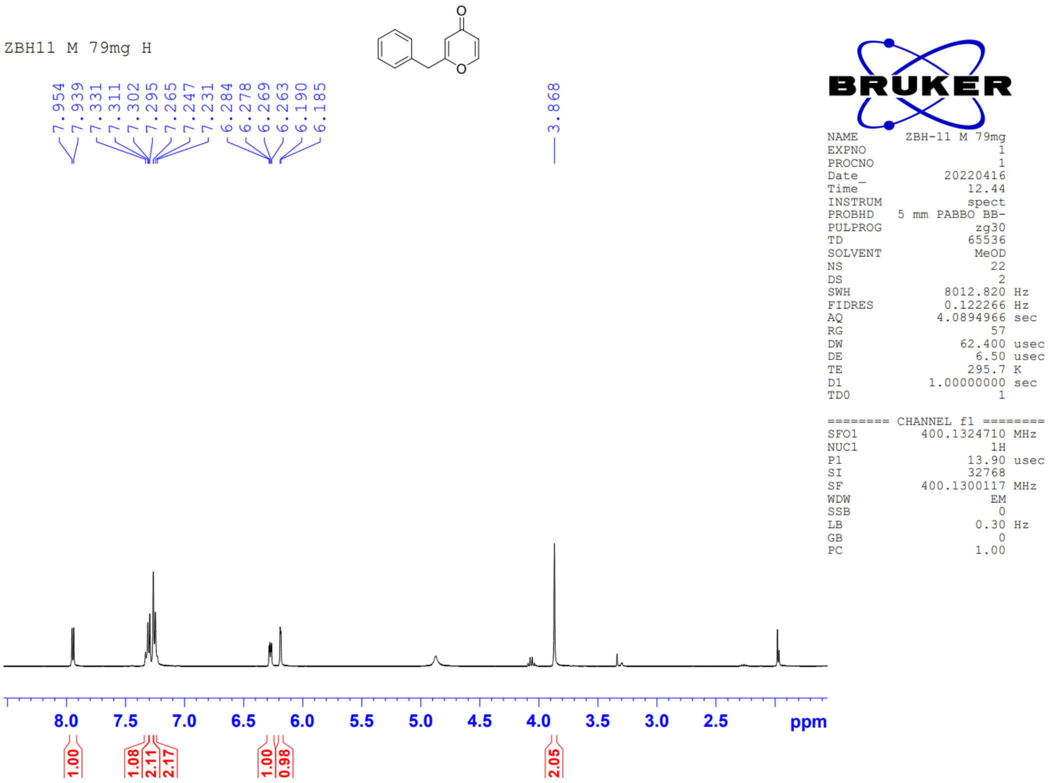

Figure S38. ^{1}H NMR spectrum of compound **13** in MeOD-d_4.

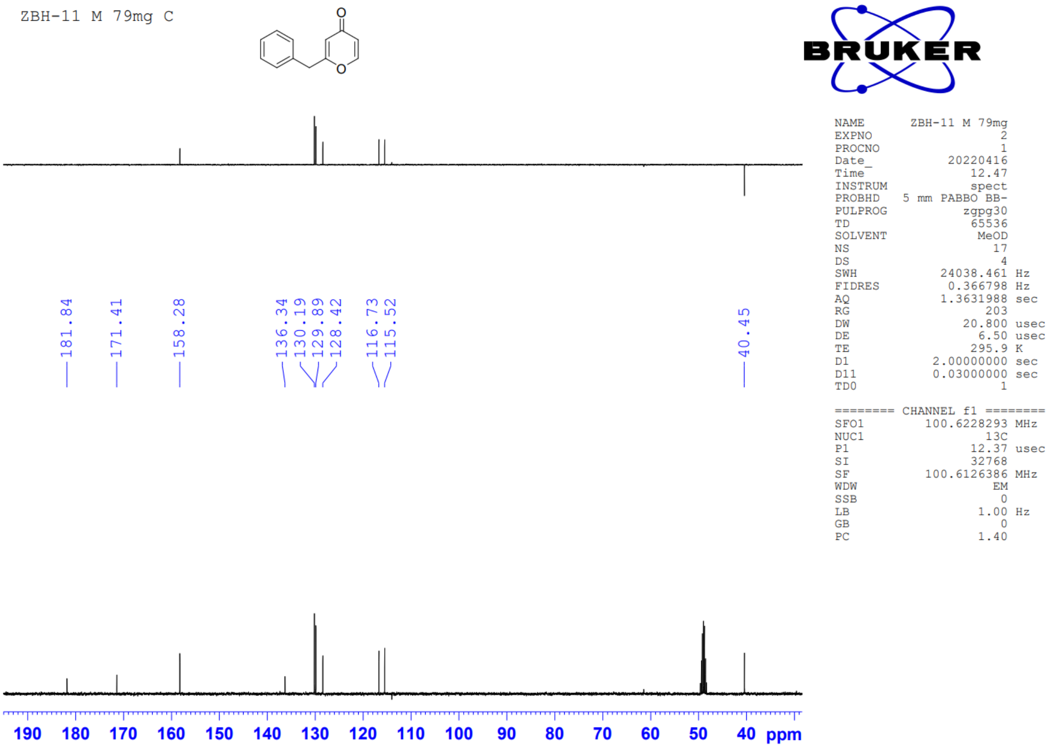

Figure S39. ^{13}C NMR spectrum of compound **13** in MeOD-*d*$_4$.

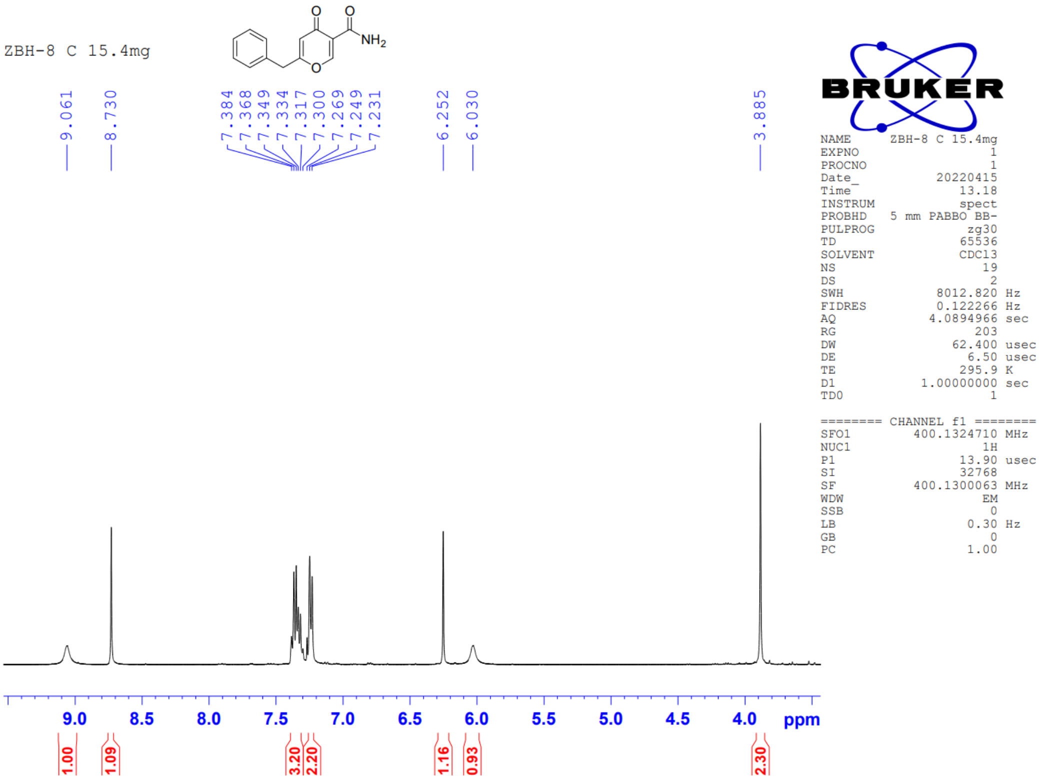

Figure S40. ^{1}H NMR spectrum of compound **14** in $CDCl_3$.

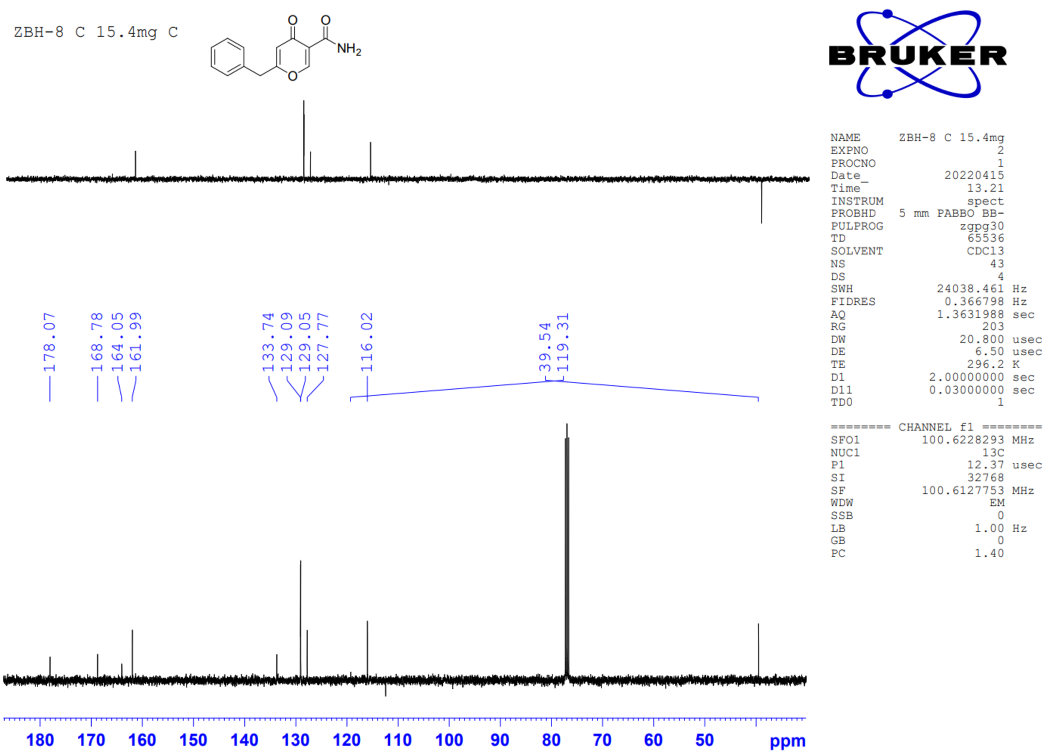

Figure S41. ^{13}C NMR spectrum of compound **14** in $CDCl_3$.

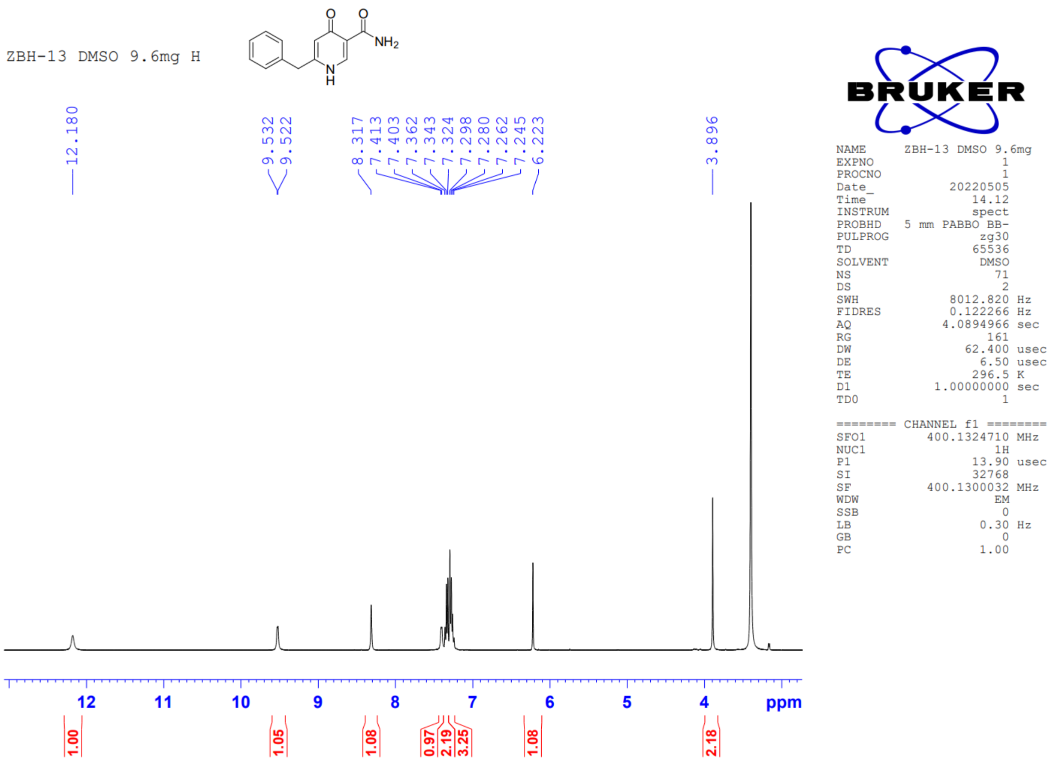

Figure S42. ^{1}H NMR spectrum of compound **15** in DMSO-d_6.

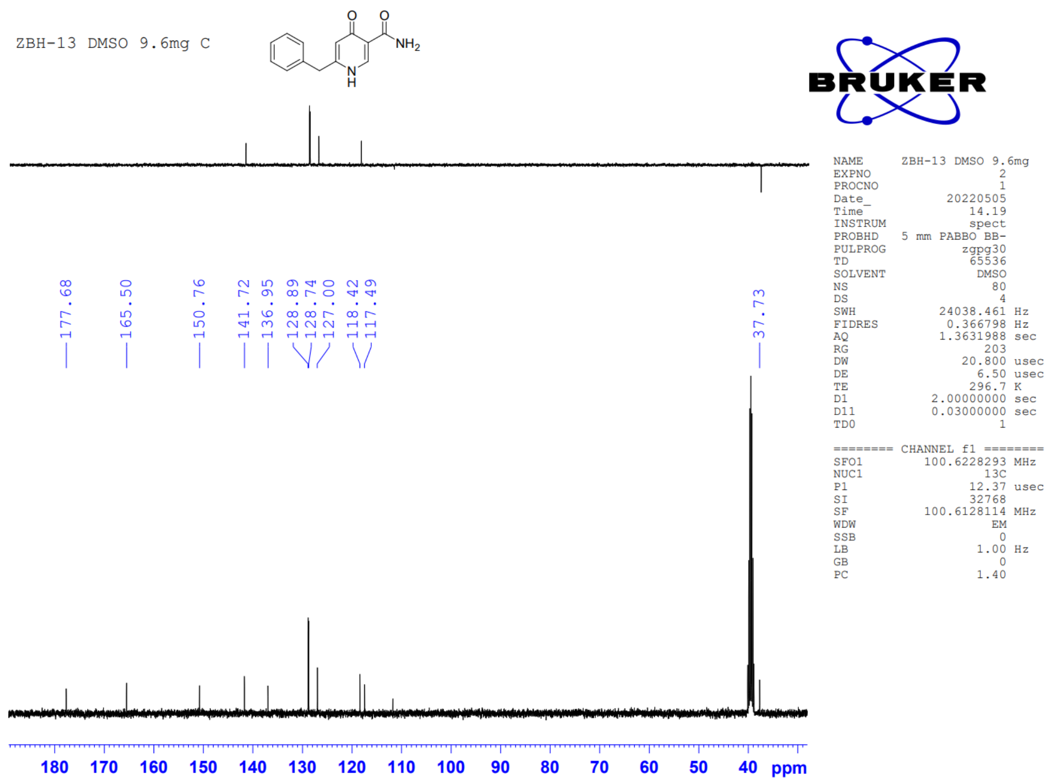

Figure S43. ^{13}C NMR spectrum of compound **15** in DMSO-d_6.

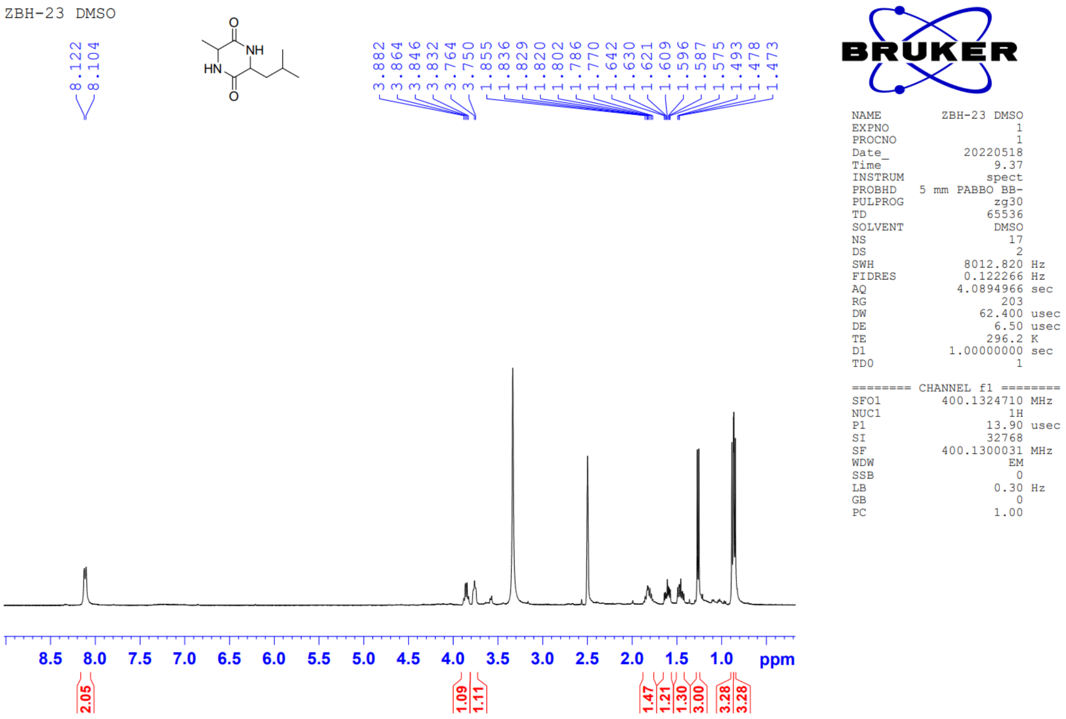

Figure S44. ^{1}H NMR spectrum of compound **16** in DMSO-d_6.

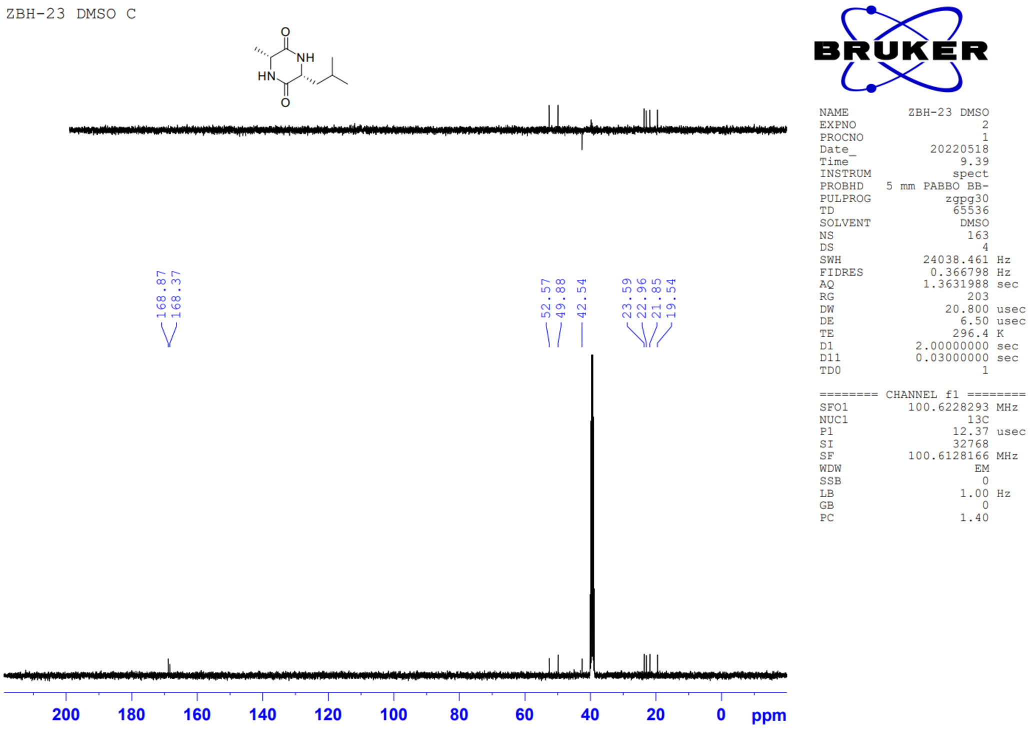

Figure S45. ^{13}C NMR spectrum of compound **16** in DMSO-d_6.

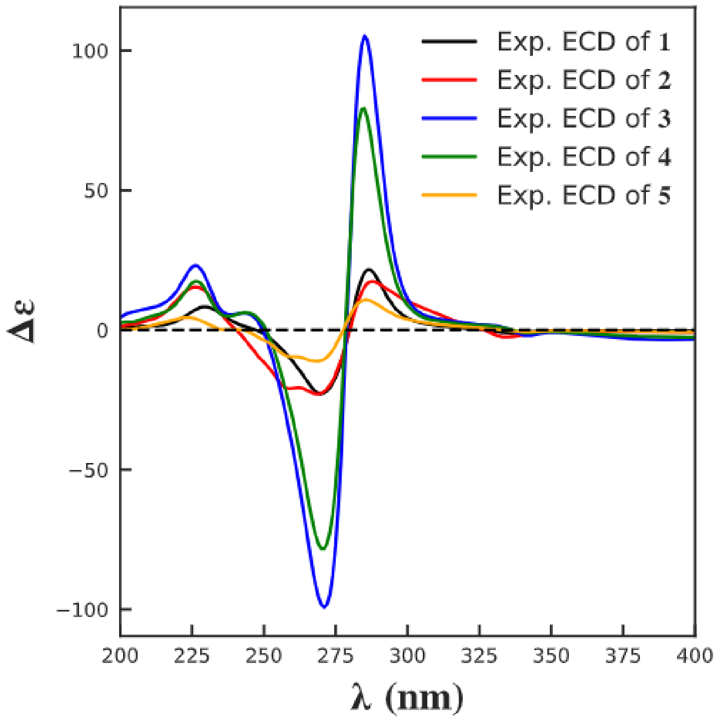

Figure S46. Experimental ECD spectra of compounds **1–5**.

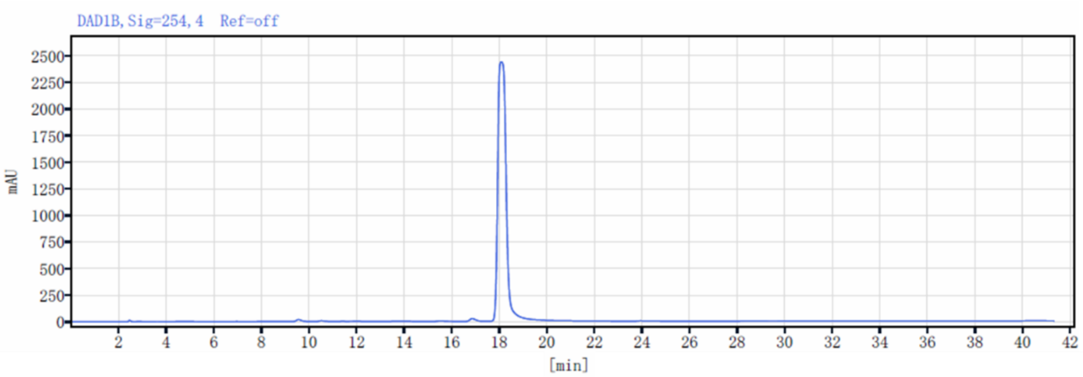

Figure S47. HPLC chromatogram of compound **1** analyzed using an OptiChiral A1 column.
